# Investigations on Organic Push–Pull Dyes for Luminescent Solar Concentrator Applications

**DOI:** 10.1021/acsaom.5c00605

**Published:** 2026-02-20

**Authors:** Roberto Bondi, Antonino Arrigo, Ejdi Cela, Luigi Vaccaro, Assunta Marrocchi, Francesco Marchini, Anna Laura Pisello, Francesco Nastasi, Loredana Latterini

**Affiliations:** † Nano4Light Lab, Department of Chemistry, Biology and Biotechnology, 9309 University of Perugia, Perugia 06123, Italy; ‡ Department of Chemical, Biological, Pharmaceutical and Environmental Sciences, and Interuniversitary Research Center for Artificial Photosynthesis (Solar Chem, Messina Node), 550300 University of Messina, V. F. Stagno d’Alcontres 31, Messina 98166 , Italy; § Laboratory of Green S.O.C., Dipartimento di Chimica, Biologia e Biotecnologie, Università degli Studi di Perugia, Via Elce di Sotto 8, Perugia 06123, Italy; ∥ Department of Engineering, 9309 University of Perugia, Perugia 06125, Italy; ⊥ EAPLAB @ CIRIAFInteruniversity Research Centre on Pollution and Environment Mauro Felli, 9309 University of Perugia, Perugia 06125 , Italy

**Keywords:** luminescent solar concentrators, organic emitters, push−pull dyes, photovoltaics

## Abstract

In recent years, the focus on luminescent solar concentrator (LSC) materials has been renewed thanks to their properties that support their integration into PV technologies in buildings and in the urban environment. In this work, three dyes bearing push–pull units and presenting anthracene (compound 1) or 2,1,3-benzothiadiazole (BTZ-P6t, compound 2, and TBTZ-P12t, compound 3) as the central chromophore module are investigated as luminophores for the LSCs based on polyacrylate. The optical and luminescence characterization of the dyes in solution and in polyacrylate panels has been carried out to examine the impact of medium polarity and stiffening on the photophysical behavior of the dyes. The photoluminescence quantum yield (PLQY), decay times, and radiative and nonradiative rate constants have been evaluated together with the overlap integral to rationalize the reabsorption phenomena. The photophysical parameters highlight that medium polarity and matrix stiffening have an impact on the photoluminescence properties. The evaluation of the photovoltaic performance, performed by placing an edge of dye panels in contact with a silicon PV device, shows that the panels act as LSCs. In particular, compound 3 exhibits the highest value of PLQY (81%), resulting in the highest value of PV light-to-energy conversion efficiencies (η_opt_%, 2.8%). This study proposes a thorough and correlated examination of the photophysical characteristics of molecular systems when the media are switched from solution to acrylate panels in order to rationalize the photovoltaic performance of the prepared LSCs. Although the prepared dye-acrylate panels fall outside accepted standard dimensions for LSC size, this study is relevant to designing chromophore architecture for enhanced efficiencies for LSCs.

## Graphical abstract

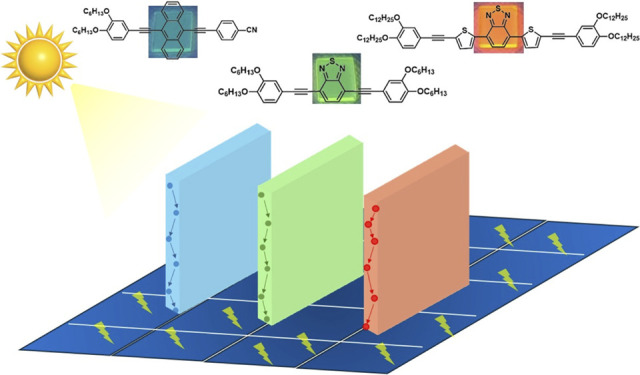

## Introduction

The climate and environmental issues caused to the *spaceship* Earth^1^ by fossil fuels, in combination with the increasing global energy demand, are powering the attention on strategies to mitigate global warming^2,3^ and driving a transition toward renewable energies in our society.^4–8^ Among these, the conversion of sunlight into electricity using photovoltaic (PV) panels is a dominant technology.^9,10^ Despite the use of PV has been diffused globally in the last decades, today less than 1% of our total energy consumption is generated by PV.^11^ To further expand the diffusion of PV panels, some habitable landscapes would be covered, implying environmental and strategic consequences.^12^ Moreover, the costs related to materials and manufacturing processes of PV devices limit the expansion of such technology worldwide. The efforts of several researchers are focused to develop the so-called Building Integrated Photovoltaics (BIPV), where PV elements are incorporated in the structure of the buildings, thus allowing an unnoticeable diffusion of the PV devices on large urban areas.^13^ Luminescent solar concentrators (LSC) are devices belonging to the BIPV family; they are transparent thin slabs containing luminophores which, once photoexcited by sunlight, emit photons that are waveguided, thanks to total internal reflection phenomena, to the edges of the slab, where PV panels are mounted and convert emitted light into electrical energy.^14–21^ The LSC appeared for the first time in the literature during the 1970s,^22–24^ but the low cost of fossil fuels in the 1980s caused a decline in the interest related to this technology. In the last years, the research on LSC has been revitalized for several reasons,^25^ including the promise of potential low fabrication costs^26^ and limited environmental costs. LSCs can be fabricated in a variety of colors and shapes^27^ and could work even when irradiated by diffused light, which appears a promising feature for applications in countries with frequent cloud coverage.^28–30^

Since the performance of LSC might encounter different limitations like an insufficient light harvesting, a limited photoluminescence quantum yield (PLQY) of the luminophore, and reabsorption processes of the emitted photons, the proper choice/design of the luminophore is necessary.^31^ A suitable chromophore should have a broad absorption spectrum together with high absorption cross sections, a large Stokes shift, and a high PLQY. A variety of emitting units have been tested for LSC, such as organic dyes, semiconducting polymers, colloidal quantum dots, semiconductor nanocrystals, and perovskite nanocrystals.^15^ Organic dyes offer the advantages to tune their optical response and their photophysical behavior as well as their dispersibility in the polymer matrices by modifying their substituents.^15^ Among organic dyes, those bearing both an electron-donating group (donor, D) and an electron-withdrawing group (acceptor, A) linked by a conjugated π-system have demonstrated to present a broad and intense absorption spectrum; the D-π-A structure leads to a strong intramolecular charge transfer (ICT) when the molecule absorbs light, which results in tunable emission and wide Stokes shifts. Different works reported the use of a push–pull organic luminophore exhibiting high optical and PV performances. In 2022, Meti et al.^32^ introduced a highly effective LSC device utilizing a novel organic fluorophore (TPP1), which incorporates tetraphenylpyrazine as an aggregation-induced emission (AIE) core symmetrically adorned with electron-rich dimethylamine (donor) and electron-deficient cyano (acceptor) moieties. Also, it has been demonstrated by Sanguineti et al.^33^ that the application of peryleneimide derivatives as luminophores that exhibit an enhanced Stokes shift, significant chemical stability, compatibility with the polymeric matrix, and high luminescence efficiency leads to the development of LSCs with a fluorescent quantum yield of 70%. Albano et al. in 2018 synthesized two push–pull fluorophore dyes based on the tris­(4-ethynylphenyl)­amine; their inclusion in the PMMA matrix leads to the creation of LSCs with a maximum optical efficiency of 6.32%.^34^

The established standards for creating an effective push–pull luminophore to be applied in LSC involve ensuring planarity and extension of the bridge that links the molecular units, a charge transfer interaction between donor and acceptor groups when excited, and incorporating bulky groups to prevent aggregation.^25,33,35,36^ Bartolini et al.^37^ reported a series of orange/red organic emitters incorporating a benzo­[1,2-b:4,5-b′]­dithiophene 1,1,5,5-tetraoxide central core as an acceptor (A) unit, linked to various donor (D) and acceptor (A′) moieties, demonstrating photonic (external quantum efficiency of 8.4 ± 0.1%) and PV (device efficiency of 0.94 ± 0.06%) performances approaching the state-of-the-art. The same group in 2024 developed a quinoxaline-based DQ emitters featuring a diaryl-quinoxaline acceptor core directly connected to two triarylamine donor moieties.^38^ The prepared LSC devices show promising efficiencies, with η dev up to 0.43%, and good photostability. Other fluorophores used for LSC applications are fluorophores with a benzo­[1,2-d:4,5-d′]­bisthiazole (BBT) heterocyclic core featuring a donor–acceptor–donor (D-A-D) configuration, with Stokes shifts large enough to minimize the self-absorption effects of the fluorophores, good optical efficiencies (5.8–7.5%), and fluorescence quantum yield.^39^

However, the luminescence efficiencies in these dyes strongly depend on the medium polarity, and the flexibility of the conjugated π-system often leads to the coexistence of different conformers,^40^ leading to a disordered ground state population. In this work, we focused our attention on three different luminophores (see Chart 1) for the LSC, namely 5-(3,4-hexyloxy-phenylethynyl),10-(4-cyano-phenylethynyl)]­anthracene (compound 1), 4,7-bis-{[m,p-bis­(hexyloxy)­phenyl]­ethynyl}-2,1,3-benzothiadiazole (BTZ-P6t, compound 2), and 4,7-bis­(5-{[m,p-bis­(dodecyloxy)­phenyl]­ethynyl}­thien-2-yl)-2,1,3 benzothiadiazole (TBTZ-P12t, compound 3). Compounds 2 and 3 bear a 2,1,3-benzothiadiazole unit; this is a commonly used acceptor building block, which can be coupled to various heteroaromatic groups, such as thiophene, a donor unit that decreases the torsion angle, increasing the coplanarity and likely forming an ordered structure in the polymer.^41^ Moreover, 2,1,3-benzothiadiazole derivatives have demonstrated to act as efficient luminophores for LSC.^30,42^ Different examples of anthracene derivatives used in LSC have been reported.^43–45^ Since the anthracene-based compounds show their strongest transition dipole moments along the molecular axis,^46,47^ controlling the physical orientation of photoluminescent dyes becomes a possible way to direct light emission, so the efficiency of an LSC can be improved and the escape cone losses can be reduced.^48,49^ Furthermore, the investigated luminophores have already shown remarkable solvatochromic effects on the emission and relevant two-photon excited fluorescence,^40,50^ both properties could be beneficial in LSC applications by reducing reabsorption phenomena and enhancing solar-light harvesting. In the present work, the luminescence behavior of three dyes has been investigated in solutions and in polyacrylate panels with the aim to rationalize the design and performance of LSC.

**Chart 1:**
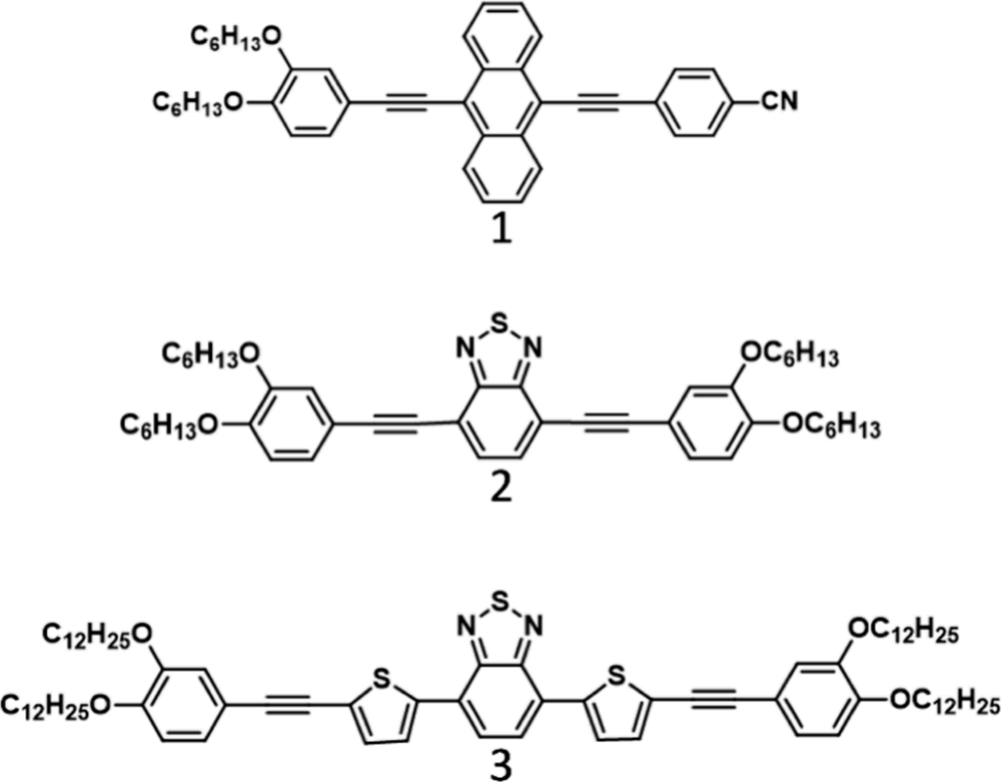
Structures of the Investigated Dyes

## Materials and Methods

### Synthesis of the Compounds

Compound 1 and compound 2 were synthesized following previously reported procedures.^37,51,52^ Compound 3 was synthesized using a slightly modified version of a published procedure.^53,54^ Further details are reported in the ESI. The structures of the compounds are reported in Chart 1.

### LSC Fabrication

Several polymeric materials have been explored to manufacture LSCs, and among these,^55^ polyacrylates appear as the most useful due to their transparency and weak absorption of UV–vis light,^10^ which is an important property to minimize the interferences of the matrix to the LSC performance.

To fabricate the LSC, the polymerization process was performed using lauryl methacrylate as the monomer and ethyl glycol dimethacrylate as the cross-linking agent, and the reaction was thermally activated using lauroyl peroxide as the initiator (see ESI).^56^ Such a reaction mixture was used to dissolve **1, 2,** and **3**, as detailed in ESI. The so-generated LSCs appear transparent and display the chromophores’ fluorescence from the borders once irradiated on the top surface (Figure 1). The LSCs have been prepared using a concentration of the chromophores of 1.3 × 10^–5^ M, which was selected as a compromise (explored concentration range 5.0 × 10^–6^ to 5.0 × 10^–5^ M) to produce a material that can display a relevant PV performance despite having a low ex coefficient. As a matter of fact, a lower dye concentration would reduce light harvesting and the solar-to-energy conversion capacity of the device, while a higher concentration would lead to strongly colored materials, which are not recommended for LSC applications, especially for smart windows. Indeed, using a 1.3 × 10^–5^ M dye concentration, a transmittance above 60–65% has been ensured in all the VIS region (see ESI).

**Figure 1:**
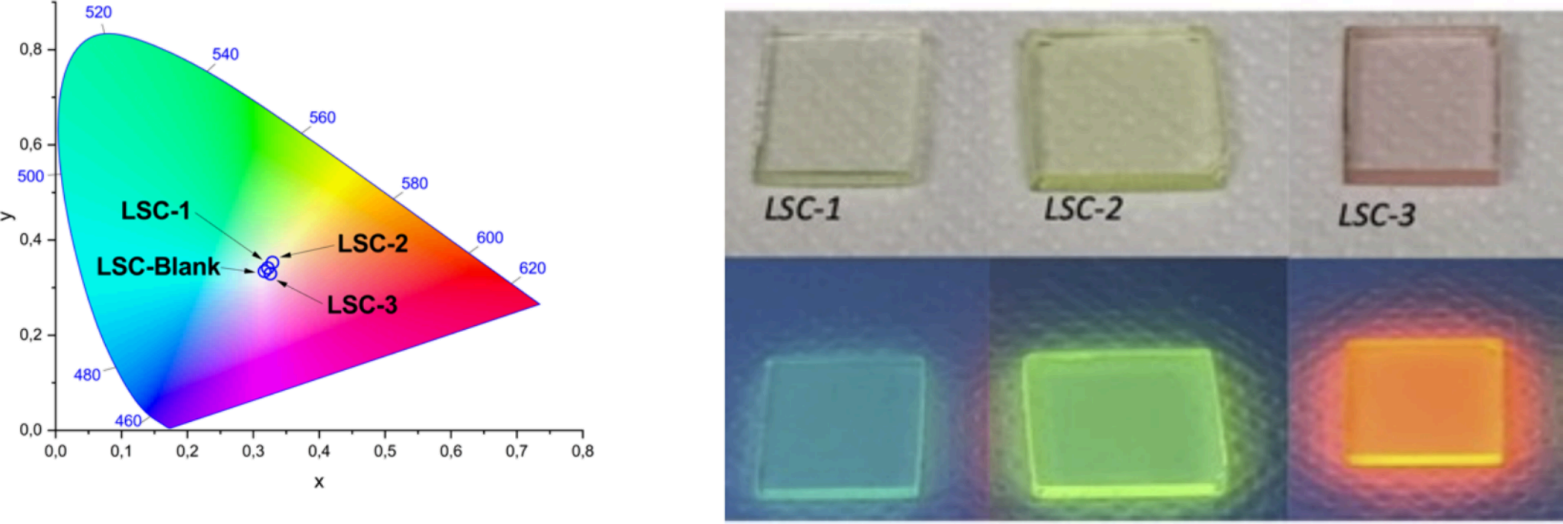
On the left: chromaticity diagram of the prepared LSC. Photograph of the LSC containing the studied chromophores before (top) and under (bottom) UV irradiation (λ = 365 nm). The panels have the following dimensions: LSC-1 1.87 × 1.78, LSC-2 1.92 × 1.89, and LSC-3 1.84 × 1.89 cm^2^ (see TableS2).

## Results and Discussion

### LSC Optical Properties

The absorption and emission spectra of the compounds under investigation in DCM and LSC are reported in Figure 2.

**Figure 2:**
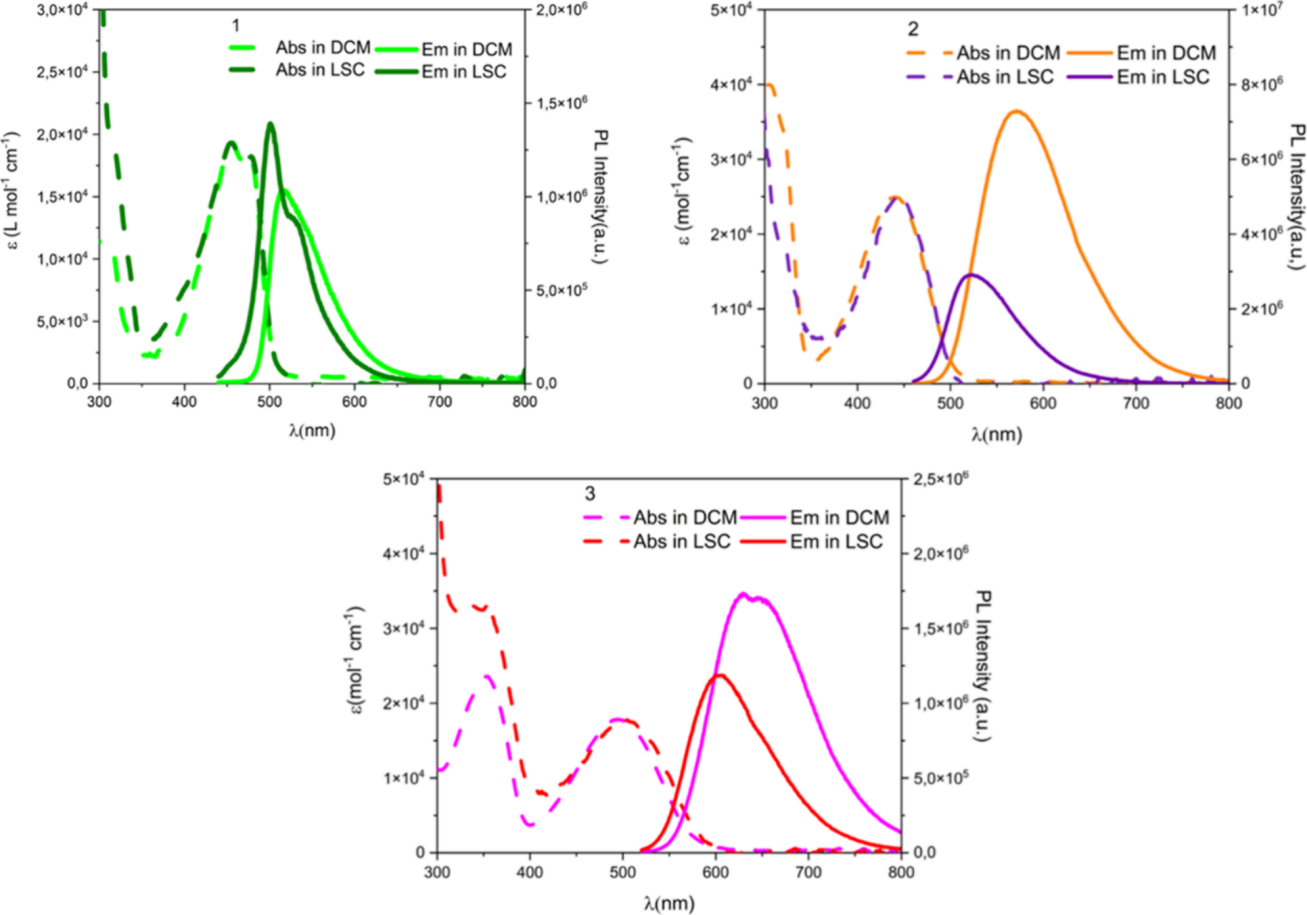
Absorption (dashed lines) and emission (full lines) spectra of the compounds in DCM and in LSC.

In DCM solutions, the three compounds present an intense absorption band in the 400–600 nm region with an absorption coefficient higher than 2 × 10^4^ M^–1^ cm^–1^ for compounds **1** and **2** and slightly lower for **3** (Table 1). The comparison of the absorption and emission spectra of the three compounds in the polyacrylate matrix versus the solution shows that in all the cases the absorption band does not change, thus suggesting that the polymerization reaction does not alter the molecular structure of the chromophores (Figure 2). Looking at the emission properties, a blue shift of the emission band is observed for all three compounds when passing from the DCM solutions to the LSC matrices. This is likely due to the different polarity of the medium; the minor polarity of the polyacrylate of the LSC induces a destabilization of the ICT character of the excited state, leading to a blue shift of the fluorescence,^40,50^ together with a reduced full width at half-maximum (fwhm) and changes of the emission band profiles (Figure 2 and Table 1), the latter particularly evident for the **LSC-1** sample.^40,50^ In general, for all the films, the fwhm reduces by about 20–25% compared to the respective solution spectra.

**Table 1:** Absorption Coefficients, Emission Maxima, Full-Width-Half Maximum (FWHM) of the Fluorescence Spectra, Overlap Integral (J), and Luminescence Quantum Efficiency (φ) of the Dyes in DCM Aerated Solution and in LSC

		λ_max_ (nm)		fwhm (nm)		J		φ (%)	
	ε (M^–1^ cm^–1^)	DCM	LSC	DCM	LSC	DCM	LSC	DCM	LSC
1	2.2 × 10^4^	515	500	71	57	14.4	25.0	37	43
2	2.4 × 10^4^	571	523	112	85	4.1	60.4	80	31
3	1.6 × 10^4^	629	600	122	101	7.3	18.2	60	81

Luminescence quantum efficiencies determined for the samples under investigation are reported in Table 1; in general, the dyes present remarkable fluorescence efficiencies, which are affected by inner-filter effects, as indicated by the overlap integral (J, integrated area between absorption and fluorescence spectra of the dyes in solution and in the LSC). In general, J has higher values for the dyes in the polymer films, indicating a larger overlap between the absorption and fluorescence spectra. In the case of compound **2**, J is more than 15 times larger in PMMA than in solution, suggesting the relevant reduction of fluorescence intensity in the panel is caused by the sample reabsorption phenomena.

The luminescence decay times of **1**, **2**, and **3** embedded in the LSC are similar to those measured in the solution phase (Table S1). Using the determined quantum efficiencies and the decay times, the rate constants for the radiative (*k*_f_) and nonradiative (*k*_nr_) deactivation processes have been calculated. The calculated values (Table S1) evidence an effect of the medium stiffening going from solution to polyacrylate panel. For **2**, *k*_f_ halved in the polyacrylate panel, while *k*_nr_ is more than 4 times higher in the film than in solution. Smaller effects are observed on *k*_f_ and *k*_nr_ values going from solution to the polymer (Table S1) for **1** and **3.**

As further information on the absorption properties of the materials, we calculated the fraction of photons absorbed, η_abs‑vis_, over the solar spectrum. The spectrum (from 370 to 1050 nm) of transmitted AM1.5G light through the LSCs was divided by the area of the solar spectrum, and the so-calculated η_abs‑vis_ is reported in Table 2 and Figure S3.

**Table 2:** Fraction of Photons Absorbed Over the Solar Spectrum (η_abs‑vis_), Color Coordinates (*x* and *y*), and Color Rendering Index (CRI) of the LSCs

LSC	η_abs‑vis_ (%)	*x*	*y*	CRI
blank	14.11	0.337	0.349	96.84
LSC-1	15.81	0.341	0.355	93.57
LSC-2	18.40	0.349	0.366	95.14
LSC-3	18.58	0.346	0.344	93.22

The values are actually not impressive compared to other results in the literature,^57^ and it is possible to observe that for LSC-blank (i.e., the polyacrylic matrix without chromophores), η_abs‑vis_ is not negligible and is attributed to some irradiation light which propagates inside the slab toward the edges. In order to understand the applicability of LSCs for installations as BIPV, the average visible transmission (AVT), color rendering index (CRI), and La*b* coordinates are calculated and reported in Table 2 and in ESI.^58^ The CRI values for all the LSCs are higher than 80 and lie in the first group of the CRI, which is appropriate for living environments (such as houses or offices). The color coordinates, calculated using the CIE 1931 chromaticity diagram (see Figure 1, and ESI for details), are placed approximately in the central region of the diagram, indicating that the materials have a reduced color intensity, which is important to keep an adequate natural illumination in indoor environments,^59^ thus allowing the use of these LSC as smart windows. The high transmittance (T%) values of the three LSC samples in the visible region, as shown in Figure S2, highlight their potential for integration into semitransparent BIPV glazing.^20^ Such optical properties also make them compatible with smart window systems, where they could be combined with other active functionalities to realize multifunctional devices.^60,61^

In order to be involved in urban architecture, an important feature for the LSCs is their photostability to continuous irradiation by sunlight. To perform photostability experiments, the upper surface of the fabricated LSCs is irradiated for 24 h using a solar simulator AM 1.5G, while a PV cell mounted at one edge of the slab detects the photocurrent every 3 h of irradiation.^62^

The results, summarized in Figure 3, show that the current intensity of the LSC-PV devices is only slightly reduced (2–4% reduction) after 24 h of continuous irradiation, pointing out that the LSC containing the organic dyes has an adequate stability.

**Figure 3:**
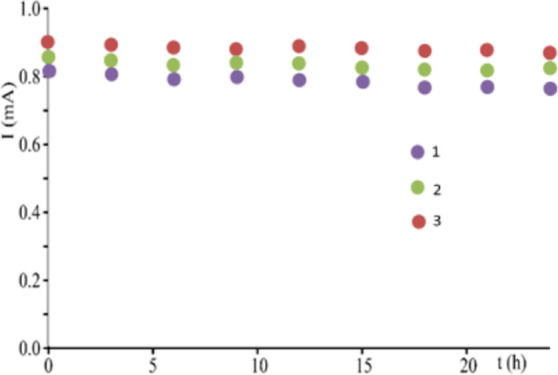
Graph illustrating the trend of the short-circuit current intensity of the fabricated LSC after 24 h of irradiation with AM 1.5G solar simulator.

### LSC PV Performance

To measure the photocurrent generated by the LSC-PV coupled system, an AM 1.5G solar simulator is used to irradiate the upper surface of the LSC, with the edge placed in contact with a silicon PV panel. The other borders of the LSC were left uncovered, while the PV cell was covered with black tape, leaving exposed only the portion necessary for the contact with the LSC, in order to reduce the contribution of diffused light to the photocurrent. It is worth to emphasize that the PV-cell mask does not alter the *J*–*V* curve of the device (Figure S5).

The optical efficiency η_opt_ of the LSC-PV was calculated according to eq 1:^58,63^

$$\eta_{\mathrm{opt}}=\frac{n^{{}^{\circ}}\;\mathrm{edge}\;\mathrm{emitted}\;\mathrm{photons}}{n^{{}^{\circ}}\;\mathrm{incident}\;\mathrm{photons}}=\frac{J_{\mathrm{LSC}}}{J_{\mathrm{PV}}\times G}$$ 1

where *J*_LSC_ (mA/cm^2^) is the short-circuit current density, obtained by dividing the short-circuit current intensity *I* (mA) by the area of the LSC in contact with the PV cell (*J* = *I*/*A*); *J*_PV_ is the short-circuit current density (mA/cm^2^) of the PV cell, obtained by dividing the current intensity (measured irradiating directly the PV cell using the AM 1.5G solar simulator) by the exposed area of the PV cell (*J*_PV_ = 15.85 mA/cm^2^ in our case); *G* is a geometrical factor, expressed by eq 2:^64^

$$G=\frac{A_{\mathrm{top}}}{2A_{\mathrm{edge}\;\mathrm{long}}\times 2A_{\mathrm{edge}\;\mathrm{short}}}$$ 2

In eq 2, *A*_top_ is the area of the top surface of the LSC slab, while *A*_edge long_ and *A*_edge short_ are the areas of the long and short borders of the LSC, respectively (the size and thickness of each LSC are reported in Table S1 in ESI).^65^ The optical efficiencies of the fabricated LSC, reported in Table 3, do not appear extremely high; nevertheless, similar values of η_opt_% are already present in the literature for LSCs based on organic dyes,^21,66^ even if the comparison with other results present in the literature is not always reliable; as a matter of a fact, the optical efficiency is strongly affected by the experimental conditions, such as the technology, the PV cell utilized, the concentration of chromophores.^67^ Since we are interested in measuring the light-harvesting ability of the devices and the efficiency of the photon-transport process within the LSCs, internal photon efficiency (η_int_) and external optical efficiency (η_ext_) are calculated according to eqs 3 and 4, respectively:^59^

$$\eta_{\mathrm{int}}=\frac{N_{\mathrm{ph}-\mathrm{out}}}{N_{\mathrm{ph}-\mathrm{abs}}}=\frac{\overset{4}{\underset{i=1}{\sum }}\int_{300}^{800}P_{i\left(\mathrm{out}\right)}\left(\lambda \right)\cdot \frac{\lambda }{hc}d\lambda }{\int_{300}^{800}P_{\mathrm{in}}\left(\lambda \right)\cdot \left(1-10^{-A\left(\lambda \right)}\right)\cdot \frac{\lambda }{hc}d\lambda }$$ 3

$$\eta_{\mathrm{ext}}=\frac{N_{\mathrm{ph}-\mathrm{out}}}{N_{\mathrm{ph}-\mathrm{in}}}=\frac{\overset{4}{\underset{i=1}{\sum }}\int_{300}^{800}P_{i\left(\mathrm{out}\right)}\left(\lambda \right)\cdot \frac{\lambda }{hc}d\lambda }{\int_{300}^{800}P_{\mathrm{in}}\left(\lambda \right)\cdot \frac{\lambda }{hc}d\lambda }$$ 4

where *N*_ph‑out_ is the total number of edge-emitted photons summed over four edges (*i* = 1–4) of the LSC, *N*_phabs_ is the total number of photons absorbed by the LSC, and *N*_ph‑in_ is the total number of photons incident on the top surface of the LSC. *N*_ph‑out_ is obtained from the sum of the output power spectra, *P*_*i*(out)(λ)_, measured for each edge of the LSC (in W nm^–1^), where λ is the wavelength of light (in nm). *P*_in(λ)_ is the input power spectrum from the solar simulator incident on the top surface of the LSC (in W nm^–1^), h is Planck’s constant (in J s), *c* is the speed of light (in m s^–1^), and *A*(λ) is the absorption spectrum of the LSC. The integrations are performed in the 300–800 nm range of the AM1.5G solar spectrum. Overall, all the LSC devices show low values of η_int_ and η_ext_ with respect to those present in the literature (Table 3).^13,64,68–74^ LSC-3 shows the highest value of η_int_ and η_ext_ with respect to the others; the maximum value of η_int_ highlights the lower probability of reabsorption processes in the LSC, as confirmed by the minor overlap between absorption and emission spectra (Figure 2), so demonstrating an optimal light-guiding ability of the emitted photons. The higher value of η_ext_, instead, is due to the higher η_abs‑vis_, as demonstrated by the better overlap of this dye’s absorption spectra with the solar emission spectrum (Figure S3).

**Table 3:** Photovoltaic Data of all the LSCs, Containing the Same Concentration of Chromophores (1.3 × 10^–5^ M) to Allow the Comparison^a^

LSC	*I* (mA)	*J*_LSC_ (mA/cm^2^)	G factor	η_opt_%	η_opt abs_ (%)	η_ext_%	η_int_%
blank	0.68	0.20 ± 0.02	1.28 ± 0.04	0.97 ± 0.10			
1	0.66	0.18 ± 0.02	1.30 ± 0.04	0.85 ± 0.10	50.51	2.6	5.2
2	0.85	0.39 ± 0.02	1.32 ± 0.04	1.86 ± 0.11	43.47	3.7	10.0
3	0.90	0.55 ± 0.02	1.24 ± 0.04	2.81 ± 0.13	61.76	3.9	13.9

a The results reported are average values of three experiments.

Generally, in LSC, a high PLQY is essential for optimizing the light concentration by decreasing the optical losses, so facilitating an effective solar photon conversion.^50,71^ Among the data reported in Table 1, it is noteworthy that for compounds 1 and 3, an increase in the PLQY is observed when the dyes are in the LSC matrix due to the conformational constraints implemented by the polymer and the ability of the matrix to stabilize the excited state, as discussed above. The highest value of PLQY, which translates into the highest value of η_opt_%, as reported for LSC-3. In LSC-3, the thiophene donor unit could help to form an ordered structure in the polymer, as indicated by the fwhm reduction, leading to one stable conformation, thus suppressing the nonradiative pathways and increasing the PLQY; from the narrowing of the emission spectrum recorded from the film sample, compared to 3 in DCM, it is possible to hypothesize that selected dye conformations are formed. The significant decrease of the PLQY in compound 2 (LSC-2) can be explained by the blue shift of the emission spectrum in LSC and the occurrence of reabsorption phenomena, as indicated by the increase of the overlap integral on going from DCM to LSC. However, the possible formation of aggregate species cannot completely be ruled out; in the case of planar ϖ-conjugated structure, the tendency to establish π–π stacking interactions results in aggregation-caused quenching of the fluorescence.^75,76^ The comparison of the fluorescence properties of compound 2 in LSC and toluene^44^ highlights the extremely good agreement of the fluorescence decay times and rate constant; this comparison indicates that the polymer matrix does not affect the dynamics of the excited states but only their energetics.

Given that the optical efficiency does not take into account the fraction of photons absorbed by the chromophores, we calculated the corrected optical efficiency η_opt,abs_ defined as^58^

$$\eta_{\mathrm{opt},\;\mathrm{abs}}\%=\frac{\eta_{\mathrm{opt}}}{\eta_{\mathrm{abs}-\mathrm{vis}\;(\mathrm{LSC}-\mathrm{chromophore})}-\eta_{\mathrm{abs}-\mathrm{vis}\;(\mathrm{BLANK})}}$$ 5

where η_abs‑vis (LSC‑chromophore)_ and η_abs‑vis (BLANK)_ are the fractions of photons absorbed by the LSC containing the chromophores and the LSC-Blank, respectively, as reported in Table 2. The values of η_opt,abs_ are collected in Table 3. The calculated values of the corrected optical efficiency (η_opt, abs_) are reported in Table 3. Notably, LSC-2 exhibits the lowest efficiency among this series of devices, primarily due to the significant increase in the overlap integral between the absorption and emission spectra when comparing the solution and LSC, while for the other samples (LSC-1 and LSC-3), absorption and emission spectra have lower spectral overlap. To investigate the quality of the waveguide inside the LSC matrix, we irradiated the top surface of the slab using a spot laser (405 nm), monitoring how the photocurrent I (detected at the LSC’s edge) is influenced by the position of the irradiation source on the upper surface. The results highlight that moving the irradiation source across the LSC top surface from one edge to the other does not considerably alter the photocurrent, indicating good quality of the waveguide inside the material. Indeed, after an initial mild decrease, the current intensity detected remained approximately constant when the optical path (meaning the distance between the laser spot on the LSC top surface and the edge in contact with the PV cell) increased. Figure 4 shows the results, where it is observable that LSC-2 displays higher values compared to those of the other chromophores. This is not surprising, considering that at 405 nm (wavelength of the laser spot), chromophore 2 absorbs more than the others of this series.

**Figure 4:**
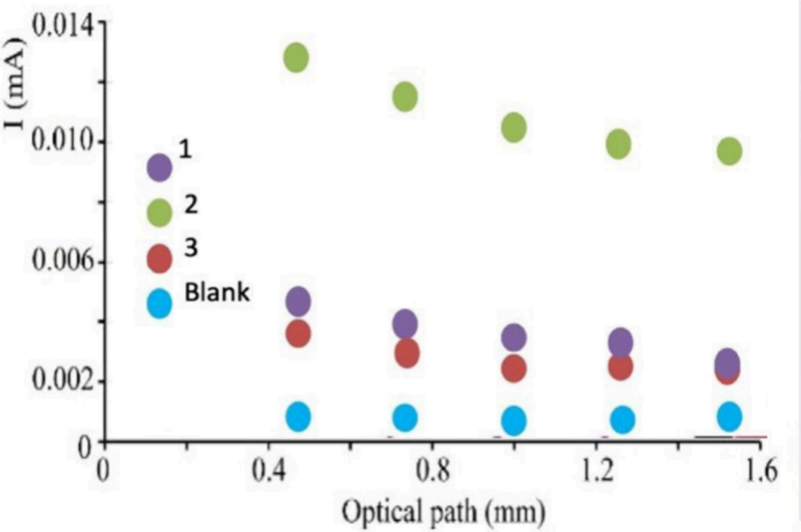
Middle graph: effect of the optical path on the photocurrent of the LSC-PV device. On the side panels, photographs of the experimental setup utilized for the cases of LSC-1 (on the left) and LSC-3 (on the right) show the light waveguiding inside the matrix.

With the intention to spotlight the contribution of the chromophores to the current of the device, external quantum efficiency (EQE%) of the LSC is obtained according to eq 6.^58^ Using the experimental setup employed for EQE measurements, the LSC-PV coupled device is placed inside the sample compartment of a spectrofluorometer, and the current intensity is detected by varying the excitation wavelength.

$${\mathrm{EQE}}_{\mathrm{LSC}}(\lambda )\%={\frac{n_{e}(\lambda )}{n_{\mathrm{il}}(\lambda )}}^{*}100$$ 6

In eq 6, *n*_e_(λ) represents the number of generated electrons by the LSC-PV system at each specific wavelength (λ), while *n*_il_(λ) denotes the number of photons striking the front surface of the LSC lightguide at the same wavelength.

The resulting EQE spectra, in the spectral range from 400 to 700 nm, are shown in Figure 5 and are essentially identical to the absorption spectra of the chromophores, thus indicating that the contribution to the photocurrent clearly rises from the excited states of the fluorophores. All the PV data, along with *J*–*V* curves and a comparison with recent results in the literature, are reported in Tables S4 and S5; Figure S4, respectively. From the comparison of PCE and η_opt_, it is noteworthy that the LSCs in this work present PCE and η_opt_ values in line with those reported in the literature.

**Figure 5:**
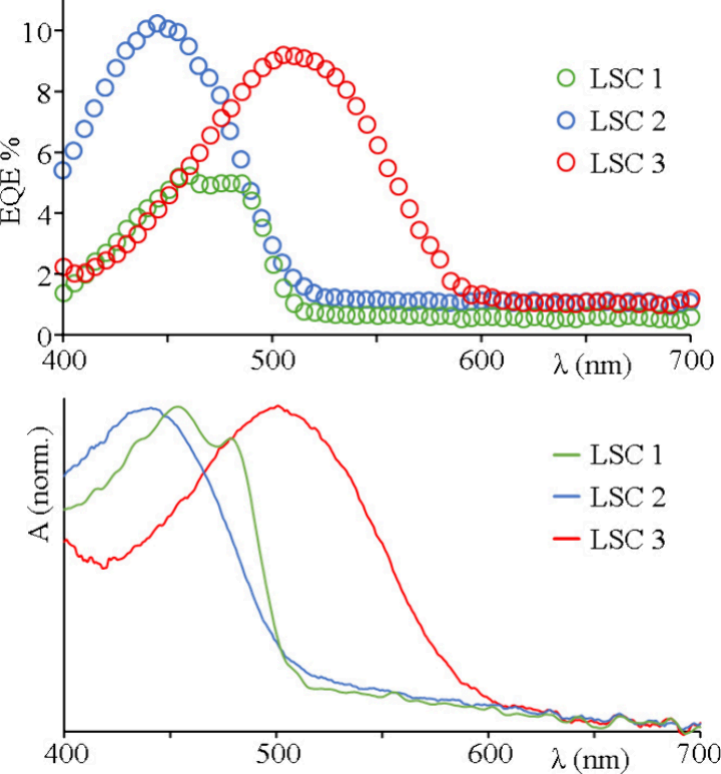
On the top panel are EQE% spectra of the fabricated LSC. On the bottom panel are absorption spectra of LSC with chromophores for comparison with EDE%. Blue for compound 2, green for compound 1, and red for compound 3.

## Conclusions

Through in-depth photophysical and PV studies, three different compounds are investigated as luminophores for the LSC, 5-(3,4-hexyloxy-phenylethynyl),10-(4-cyano-phenylethynyl)]­anthracene (compound 1), 4,7-bis-{[m,p-bis­(hexyloxy)­phenyl]­ethynyl}-2,1,3-benzothiadiazole (BTZ-P6t, compound 2), and 4,7-bis­(5-{[m,p-bis­(dodecyloxy)­phenyl]­ethynyl}­thien-2-yl)-2,1,3-benzothiadiazole (TBTZ-P12t, compound 3). To the best of our knowledge, this is the first time that these compounds have been employed as luminophores for LSC applications. The photophysical behavior of the three luminophores is deeply analyzed to find how the features of the push–pull dyes influence LSC performances measured through PV efficiency. Even though the manufactured dye-acrylate panels do not conform to the recognized standard sizes for LSCs, by having dimensions of about 2 × 2 cm^2^ instead of the standard LSC dimensions of 5 × 5 cm^2^, this research is pertinent to developing chromophore structures aimed at improving efficiencies for LSCs.

By comparing absorption and emission spectra of the three compounds in solution and inside the LSC matrix, based on polyacrylate, it can be noted that the absorption band does not change, while in the emission spectra, all three compounds exhibit a blue shift when passing from the DCM solutions to the LSC matrix. Regarding compounds 1 and 3, the PLQY increases when the chromophores are in the LSC matrix, while in the case of compound 2, a decrease is observed. The AVT, CRI, and high transmittance values, together with the L a*b* coordinates, demonstrate that the materials are not strongly colored, making them useful for illumination in indoor or outdoor environments, also paving the way for their use as smart windows. The LSCs present good photostability, with the current intensity of the LSC-PV cell devices only slightly reduced after 24 h of continuous irradiation. Incident photon conversion efficiency (IPCE) spectra and absorption spectra, in the spectral range from 400 to 700 nm, overlap, thus suggesting that the contribution to photocurrent is distinctly derived from the excited states of the fluorophores. The LSCs present an optical efficiency (η_opt_%) of 0.85, 1.86, and 2.86 for LSC-1, LSC-2, and LSC-3, respectively. Although the overall optical efficiencies of the LSC may not seem particularly high, this work suggests an extensive and integrated analysis of the photochemical and PV properties of the fluorophores proposed for the LSC, which is helpful in suggesting an enhanced general design of the chromophores for LSCs.

## Supplementary Material



## Notes

Description of the synthesis of the compounds 1,2,3; description of the fabrication of the LSC; values of luminescence decay times, kinetic constants of fluorescence (*k*_f_), and nonradiative processes (*k*_nr_) of the three compounds in DCM and in LSC; transmittance spectra of LSCs: Blue for LSC-Blank, green for LSC-4, light blue for LSC-6, and red for LSC-3; blue spectrum in each panel is the solar spectrum using the AM 1.5G filter; the red spectra represent the spectrum of transmitted AM1.5light through LSCs; L, l, and t of the fabricated LSC, used to calculate the G factor; also, the area of the LSC’s border in contact with the PV cell used to calculate J_LSC_; current intensity, exposed area and current intensity of the PV cell under direct illumination by the AM 1.5 G source; values of optical power density of the lamp and J of the various LSCs at different wavelengths (range from 400 to 700 nm); photovoltaic data (Jsc, Voc, FF, PCE%, η_op_% EDEmax%) of all the LSCs fabricated; *J*–*V* curves of the LSC systems; overlap between the emission spectra of the LSCs and the absorption spectrum of the polycrystalline silicon photovoltaic panel; ^1^H NMR spectra and ^13^C NMR spectra of compounds 1,2, and 3; comparison of the efficiency parameters of LSC–PV devices obtained in this work with those reported in the literature; and average visible transmission (AVT), color coordinates (LAB), color rendering index (CRI), and chromaticity coordinates (PDF)
