# Supplementary material for: Investigations on Organic Push–Pull Dyes for Luminescent Solar Concentrator Applications
Source: ACS Appl Opt Mater. 2026 Feb 20;4(3):775–84. doi: 10.1021/acsaom.5c00605 (PMC13036777; doi:10.1021/acsaom.5c00605)
Supplement: Supplementary file 1 [file ot5c00605_si_001.pdf]

**Supporting Information**

# Investigations on organic push-pull dyes for luminescent solar concentrator applications

Roberto Bondi[a], Antonino Arrigo[b], Ejdi Cela[c], Luigi Vaccaro[c], Assunta Marrocchi[c], Francesco Marchini[a], Anna Laura Pisello[d], Francesco Nastasi[b], and Loredana Latterini[a*].

[a]Nano4Light Lab, Department of Chemistry,Biology and Biotechnology, University of Perugia, 06123 Perugia, Italy

[b]Department of Chemical, Biological, Pharmaceutical and Environmental Sciences, and Interuniversitary Research Center for Artificial Photosynthesis (Solar Chem, Messina Node), University of Messina, V. F. Stagno d'Alcontres 31, Messina, 98166 Italy1

[c] Laboratory of Green S.O.C., Dipartimento di Chimica, Biologia e Biotecnologie, Università degli Studi di Perugia, Via Elce di Sotto 8, 06123 Perugia, Italy

[d] Department of Engineering, University of Perugia, 06125 Perugia, Italy; EAPLAB @ CIRIAF Interuniversity Research Centre on Pollution and Environment Mauro Felli., University of Perugia, 06125 Perugia, Italy

* Corresponding author: Loredana Latterini – loredana.latterini@unipg.it

## Materials and methods

### Chemicals

The solvents and chemicals were provided from commercial chemical suppliers (Merck). The monomer and cross-linker of the polymerization reaction were purified with basic activated alumina to remove the inhibitor of the polymerization, present in these reagents.

Ethylene glycol dimethacrylate 98%, lauroyl peroxide for synthesis, and lauryl methacrylate 96% (all of them purchased from Sigma-Aldrich) were used as reagents for LSC fabrication.

### Synthesis of compounds 1, 2 and 3

### Synthesis of compound 1

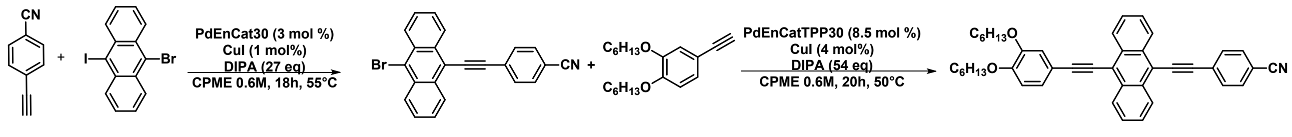


4a 4b 5 6 1

**Scheme S1.** Synthesis of compound **1**

The compound was synthesized following the procedure described by Bartollini et al.[1] In a screw capped vial equipped with a magnetic stirrer 4-ethynyl-benzonitrile (**4a**) (0.055 g, 0.43 mmol), was combined with degassed cyclopentyl methyl ether (CPME, 0.66 mL), 9-iodo-10-bromo-anthracene (**4b**, 0.149 g, 0.39 mmol), diisopropylamine (**DIPA**, 1.063 g, 10.53 mmol), CuI (0.00074 g, 0.039 mmol), and PdEnCat30 (0.029 g, 0.011 mmol). The resulting mixture was stirred vigorously at 55°C for 8h. After completion of the reaction, the mixture was diluted with 2 mL of $CHCl_3$, the catalyst was removed by filtration, and the solvent was evaporated under reduced pressure to give 4-[(10-

Bromo-9-anthryl)ethynyl]benzonitrile (**5**). This latter was purified by flash silica gel chromatography (ETP:DCM 9:1, v:v) to obtain yellow crystals. Isolated yield: 85% (127 mg).

In a screw capped vial equipped with a magnetic stirrer 3,4-bis(hexyloxy)ethynylbenzene (**6**) (0.130 g, 0.43 mmol), was combined with degassed cyclopentyl methyl ether (CPME, 0.66 mL), 4-[(10-Bromo-9-anthryl)ethynyl]benzonitrile (**5**, 0.39 mmol), diisopropylamine (**DIPA**, 2.126 g, 21.06 mmol), CuI (0.0029 g, 0.015 mmol), and PdEnCatTPP30 (0.082 g, 0.033 mmol). The resulting mixture was stirred vigorously at 50°C for 20h. After completion of the reaction, the mixture was diluted with 2 mL of $CHCl_3$, the catalyst was removed by filtration, and the solvent was evaporated under reduced pressure to give 4-[(10-{[3,4-bis(hexyloxy)phenyl]ethynyl}-9-anthryl)ethynyl]benzonitrile (**1**). This latter was purified by recrystallization from ethyl acetate (2x25 mL) yielding red-orange crystals of the pure compound. Isolated yield: 70% (165 mg). $^1$H-NMR ($CDCl_3$, δ): 8.71 (m, 2H), 8.62 (m, 2H), 7.86 (d, $J$ = 8.3 Hz, 2H), 7.75 (d, $J$ = 8.1 Hz, 2H), 7.66 (m, 4H), 7.36 (m, 1H), 6.94 (m, 1H), 4.09 (dt, $J$ = 12.6, 6.6 Hz, 4H), 1.87 (m, 4H), 1.38 (m, 8H), 1.25 (m, 4H), 0.93 (m, 6H). 13C-NMR (CDCl3, δ):149.0, 132.4, 132.2, 132.0, 131.8, 128.3, 127.5, 127.2, 126.8, 126.7, 125.4, 116.7, 115.2, 113.3, 69.5, 69.2, 31.6, 29.7, 29.2, 29.1, 25.7, 22.6, 14.0

**Synthesis of compound 2**

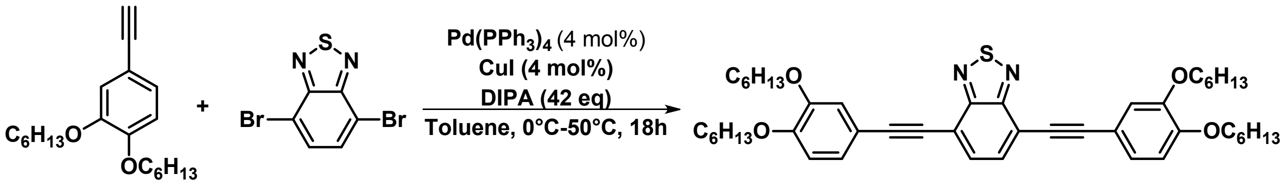


7a 7b 2

**Scheme S2.** Synthesis of compound **2**.

The compound was synthesized following the procedure previously described by Silvestri et al.[2] A 25 mL round-bottom flask was charged with dry toluene (6 mL), 4,7-dibromo 2,1,3-benzothiadiazole

(**7b**, 0.15 g, 0.51 mmol), CuI (0.004 g, 0.02 mmol), $Pd(PPh_3)_4$ (0.025 g, 0.02 mmol), and diisopropylamine (**DIPA**, 3 mL, 21.41 mmol). The reaction mixture was degassed by purging with argon at 0 °C. Subsequently, 3,4-bis(hexyloxy)ethynylbenzene (**7a**, 0.33 g, 1.1 mmol) was added, and the mixture was heated to 50 °C for 18 h. After completion, the solvent was removed under reduced pressure. The resulting crude product was purified via silica gel column chromatography (ETP:DCM 3:2, v:v). This afforded 4,7-bis-{[m,p-bis(hexyloxy)phenyl]ethynyl}-2,1,3-benzothiadiazole (**2**) as yellow crystals. Isolated yield: 80% (301 mg). $^1$H NMR ($CDCl_3$): δ): 7.75 (s, 2H), 7.25 (m, 1H), 7.23 (m, 1H), 7.16 (m, 2H), 6.87 (m, 2H), 4.03 (m, 8H), 1.84 (m, 8H), 1.35 (m, 16H), 0.86-0.19 (m, 12 H).; 13C NMR (CDCl3, δ): 154.5, 150.5, 148.9, 132.3, 125.8, 117.2, 116.9, 114.2, 113.2, 98.2, 84.1, 69.4, 69.2, 31.7, 29.3, 29.2, 25.8, 22.7, 14.1.

## Synthesis of compound 3

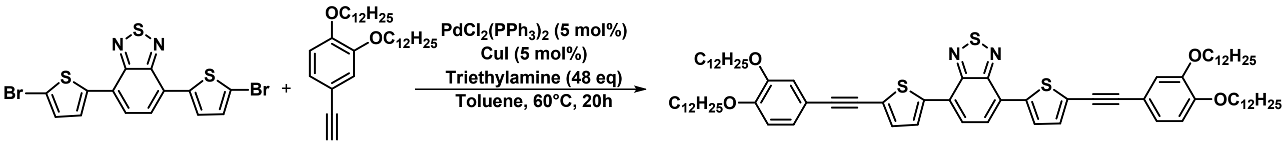


8 9 3

**Scheme S3.** Synthesis of compound **3**.

The compound was synthesized following the procedures described by Marrocchi[3] and Wu[4]. To a two-necked round-bottom flask, equipped with a nitrogen inlet, compound **9** (0.847 g, 1.8 mmol), 4,7-bis(5-bromo-2-thienyl)-2,1,3-benzothiadiazole (**8**, 0.340 g, 0.75 mmol), CuI (0.007 g, 0.04 mmol), and $PdCl_2(PPh_3)_2$ (0.027 g, 0.04 mmol) were added. The flask was flushed with nitrogen, and anhydrous toluene (10 mL) and dry triethylamine (5 mL) were injected via syringe. The reaction mixture was stirred at 60°C for 20h under a nitrogen atmosphere. After completion, the solvent was removed under reduced pressure, and the residue was purified by column chromatography on silica gel (Hex:EtOAc 1:1, v/v) yielding 4,7-bis(5-{[m,p-bis(dodecyloxy)phenyl]ethynyl}thien-2-yl)-

2,1,3-benzothiadiazole, as a red solid powder. Isolated yield: 19% (176 mg). $^1$H-NMR ($CDCl_3$, δ): 8.03 (d, J = 4.0 Hz, 2H), 7.88 (s, 2H), 7.32 (d, J = 3.9 Hz, 2H), 7.11 (d, J = 8.2, 2H), 7.05 (d, J = 1.9 Hz, 2H), 6.85 (d, J = 8.3 Hz, 2H), 4.02 (t, J = 6.6 Hz, 8H), 1.88-1.79 (m, 8H), 1.48-1.27 (m, 72H), 0.90-0.86 (m, 12H). 13C-NMR (CDCl3, δ):140.0, 132.3, 127.46, 125.63, 125.57, 125.31, 124.9, 116.4, 113.3, 95.64, 89.54, 69.3, 69.2, 31.9, 29.71, 29.67, 29.65, 29.63, 29.42, 29.38, 29.22, 29.20, 26.03, 26.01, 22.7, 14.12.

**Characterization**

A Jasco V-560 spectrophotometer was utilized for UV/Vis absorption measurements both in liquid and in solid state. A Jobin Yvon-Spex Fluoromax P spectrofluorimeter, equipped with a Hamamatsu R3896 photomultiplier, was utilized for luminescence measurements both in liquid and in solid state. The emission spectra were corrected for the photomultiplier response using a program purchased with the fluorimeter. To measure the luminescence decay times, an Edinburgh OB 900 time-correlated single-photon-counting spectrometer was used, equipped with a Hamamatsu PLP 2 laser diode (59 ps pulse width at 408 nm) as excitation source. The estimated experimental errors on the molar absorption coefficient and luminescence lifetime is 5%; the estimated errors on the absorption and emission band maximum are 2 nm; the estimated error on the luminescence quantum yield is 10%. The quantum yield of the LSC materials was measured with a Quantum Efficiency Sphere 3 coated in special BaSO4 paint by StellarNet Inc, equipped with a SL1-LED with 5V DC Out.

**Fabrication of LSC**

A thermal polymerization route was followed to prepare the luminescent solar concentrators. Lauryl methacrylate (LMA) acted as monomer, ethyl glycol dimethacrylate (EGDM) as cross-linking agent, and lauroyl peroxide as initiator. The ratio of these reagents was: LMA 65% w/w; EGDM 35% w/w; lauroyl peroxide 0.65% w/w with respect to LMA.[5,6]

The monomer LMA was used to dissolve the initiator and the mixture was heated at 80°C stirring under air condition, for 30 minutes. Then the cross-linking agent was added, keep stirring at room temperature for additional 10 minutes. The so-obtained mixture was used to dissolve each chromophore and it was left in the ultrasonic bath for 10 minutes. Finally, the mixture was introduced into a "homemade" stamp as shown in **Figure S1**, and placed in an oven at 90°C for 40 minutes.

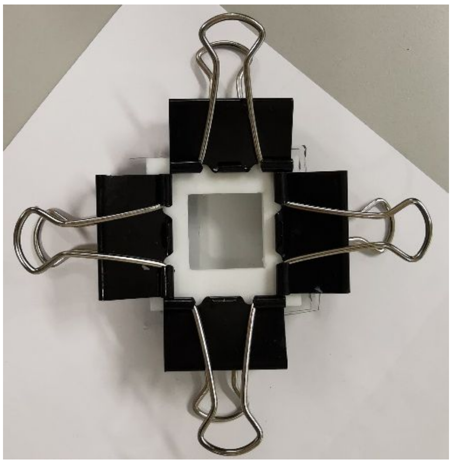

**Figure S1**. Homemade stamp for LSCs, composed by two glasses separated by a teflon support having a hole to introduce the mixture, and clamped firmly together. The dimensions of the stamp are 2x2 $cm^2$.

At the end of the polymerization process, the fluid solution turns into a solid LSC slab, which was treated by sanding the surfaces and the borders to remove defects, making the material smoother. The same procedure was followed multiple times per each chromophore, using different concentrations.

**Table S1.** Values of luminescence decay times, kinetic constants of fluorescence ($k_f$) and non-radiative processes ($k_{nr}$) of the three compounds in DCM and in LSC.

| Compound | τ (ns) | | $k_f$ ($s^{-1}$) x $10^{-8}$ | | $k_{nr}$ ($s^{-1}$) x $10^{-8}$ | |
|---|---|---|---|---|---|---|
| | in DCM | in LSC | in DCM | in LSC | in DCM | in LSC |
| **1** | 2.8 | 3.0 | 1.3 | 1.4 | 2.2 | 1.9 |
| **2** | 5.9 | 5.0 | 1.4 | 0.7 | 0.3 | 1.4 |

| | | | | | | |
|---|---|---|---|---|---|---|
| 3 | 4.9 | 4.5 | 1.2 | 1.8 | 0.8 | 0.4 |

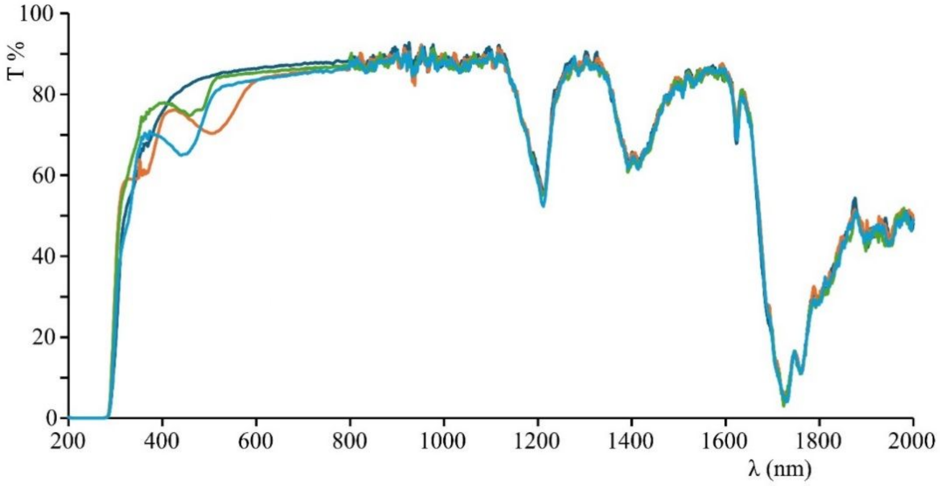


**Figure S2.** Transmittance spectra of LSCs: Blue for LSC-Blank, green for LSC-4, light-blue for LSC-6 and red for LSC-3.

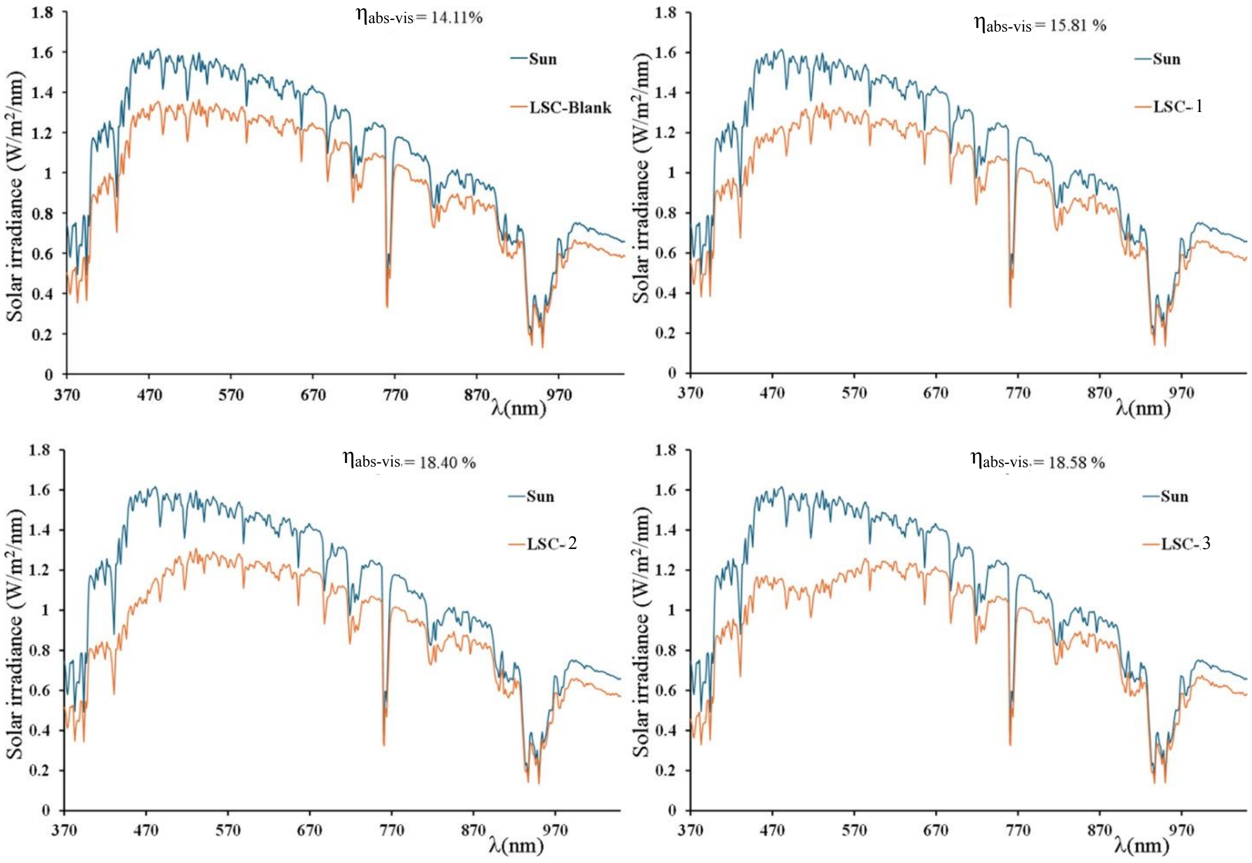

**Figure S3.** The blue spectrum in each panel is the solar spectrum using the AM 1.5G filter. The red spectra represent the spectrum of transmitted AM1.5light through LSCs

**Photovoltaic investigations**

To investigate on the photovoltaic performances, one edge of the LSC was placed in direct contact with a polycrystalline silicon photovoltaic leaving the other 3 edges of the slab uncovered. The photovoltaic (PV) panel used consists of 4 cells connected in parallel, the main electrical characteristics of the panel are: open-circuit voltage (Voc) of 0.55 V and short-circuit current (Isc) of 100 mA.

A solar simulator, consisting in a Xenon lamp with an AM 1.5 Global filter generating UV-vis light with the power intensity of 1 Sun (100 mW $cm^{-2}$), was used to irradiate perpendicularly the surface of the LSC and photocurrent was measured using a digital multimeter Agilent 34401A.[7–10]

The G-factor is calculated according to Eq. S1:[11]

$$G = \frac{A\ top}{2A\ edge\ long + 2A\ edge\ short} = \frac{L\ x\ l}{2\ (L\ x\ t) + 2\ (l\ x\ t)} \qquad \textbf{Eq. S1}$$

where *L* and *l* are the long and the short sides respectively, and *t* is the thickness of the slab. The sizes of all the LSCs are reported in **Table S1**.

**Table S2**. Length (L), width (l) and thickness (t) of the fabricated LSC, used to calculate the G factor according to **Eq. S1**; the area of the LSC's border in contact with the PV cell (used to calculate $J_{LSC}$) is also reported.

| LSC | *L (cm)* | *l (cm)* | *t (cm)* | *G factor* | *Contact area ($cm^2$)* |
|---|---|---|---|---|---|
| Blank | 1.86 | 1.73 | 0.35 | 1.28 | 0.651 |

| | | | | | |
|---|---|---|---|---|---|
| 1 | 1.87 | 1.78 | 0.35 | 1.30 | 0.6545 |
| 2 | 1.92 | 1.89 | 0.36 | 1.32 | 0.6804 |
| 3 | 1.84 | 1.56 | 0.34 | 1.24 | 0.6256 |

$J_{PV}$, mentioned in Eq.1 in the main manuscript, was calculated dividing the short circuit current intensity of the PV cell (measured irradiating directly the PV cell using the solar simulator) by the exposed area of the panel (**Table S2**).

**Table S3**. Current intensity, exposed area and current intensity of the PV cell under direct illumination by the AM 1.5 G source.

| | **Area PV (cm$^2$)** | **J $_{PV}$ (mA/cm$^2$)** | **V$_{oc}$ (V)** | **FF** |
|---|---|---|---|---|
| Masked PV Cell | 0.94 | 15.85 | 0.57 | 67.80 |
| PV Cell | 3.35 | 16.23 | 0.58 | 62.43 |

The total active area of the photovoltaic panel is approximately 3.35 cm$^2$. However, a mask was applied during measurements to expose only the region in direct contact with the LSC, limiting the effective area to ~0.9 cm$^2$. This reduced area accounts for the relatively low current density values reported in Table S2.

**Photocurrent measured by varying the optical path**

One edge of the LSC was placed in contact with the PV, while the slab surface was irradiated by a spot laser at λ = 406 nm. The LSC was put on a platform which allowed to control the position of the laser spot on the slab surface. The photocurrent was measured with the same equipment mentioned above.

**Fraction of photons absorbed over the solar spectrum**

The fraction of photons absorbed of each LSC material over the solar spectrum ($\lambda_{abs}$ = 370-1050 nm) was calculated as follows:

$$\eta_{\text{abs-vis}} = \frac{\int_{\lambda=370\,nm}^{\lambda=1050\,nm} I_0 - I_T}{\int_{\lambda=370\,nm}^{\lambda=1050\,nm} I_0} \quad \textbf{Eq.S2}$$

## External quantum efficiency (EQE)

For the experiments to calculate the EQE, the LSC-PV coupled device was placed in the sample older of a Jobin Yvon-Spex Fluoromax P used as irradiation source. The short-circuit current intensity was measured with a digital multimeter Agilent 34401A. The optical power density of the irradiation source was measured using a power-meter by Newport (model 843-R equipped with a calibrated photodetector 818-SL, working in the range from 400 nm to 1100 nm).

## Power conversion efficiency (PCE)

The power conversion efficiency (PCE) of the LSC-PV systems was calculated from the photovoltaic parameters listed in Table S4, using the following equation:

$$PCE = \frac{J_{sc} * V_{oc} * FF}{P_0} \quad \textbf{Eq.S3}$$

where, $J_{SC}$ is the short-circuit current density, $V_{OC}$ is the open-circuit voltage, FF is the fill factor, $P_0$ is the power density of the AM 1.5G solar spectrum as the standard input intensity

**Table S4.** Values of optical power density of the lamp and J of the various LSC at different wavelengths (range from 400 nm to 700 nm).

| λ (nm) | P mW/cm$^2$ | J LSC-Blank (mA/cm$^2$) | J LSC-1 (mA/cm$^2$) | J LSC-2 (mA/cm$^2$) | J LSC-3 (mA/cm$^2$) |
|---|---|---|---|---|---|
| 400 | 0.356 | 0.000297 | 0.001637 | 0.006232 | 0.002640 |
| 405 | 0.354 | 0.000281 | 0.002039 | 0.007011 | 0.002413 |
| 410 | 0.366 | 0.000314 | 0.002488 | 0.008230 | 0.002545 |
| 415 | 0.368 | 0.000347 | 0.003050 | 0.009200 | 0.002847 |
| 420 | 0.371 | 0.000429 | 0.003483 | 0.010229 | 0.003167 |
| 425 | 0.359 | 0.000380 | 0.003852 | 0.010832 | 0.003356 |

| | | | | | |
|---|---|---|---|---|---|
| 430 | 0.351 | 0.000495 | 0.004334 | 0.011390 | 0.003695 |
| 435 | 0.353 | 0.000545 | 0.004880 | 0.011993 | 0.004167 |
| 440 | 0.353 | 0.000578 | 0.005297 | 0.012610 | 0.004770 |
| 445 | 0.345 | 0.000545 | 0.005634 | 0.012684 | 0.005185 |
| 450 | 0.378 | 0.000661 | 0.006645 | 0.013815 | 0.006373 |
| 455 | 0.359 | 0.000677 | 0.006934 | 0.013139 | 0.006863 |
| 460 | 0.388 | 0.000760 | 0.007624 | 0.013683 | 0.008088 |
| 465 | 0.447 | 0.000875 | 0.008427 | 0.014830 | 0.010124 |
| 470 | 0.421 | 0.000859 | 0.007929 | 0.013492 | 0.010558 |
| 475 | 0.325 | 0.000817 | 0.006292 | 0.009818 | 0.008937 |
| 480 | 0.327 | 0.000677 | 0.006404 | 0.008510 | 0.009521 |
| 485 | 0.314 | 0.000677 | 0.006180 | 0.007099 | 0.009898 |
| 490 | 0.313 | 0.000710 | 0.005522 | 0.005864 | 0.010483 |
| 495 | 0.309 | 0.000694 | 0.004414 | 0.004747 | 0.010916 |
| 500 | 0.293 | 0.000694 | 0.002809 | 0.003513 | 0.010747 |
| 505 | 0.295 | 0.000710 | 0.001926 | 0.002866 | 0.011124 |
| 510 | 0.294 | 0.000694 | 0.001332 | 0.002307 | 0.011161 |
| 515 | 0.292 | 0.000694 | 0.001027 | 0.001969 | 0.011086 |
| 520 | 0.287 | 0.000694 | 0.000979 | 0.001690 | 0.010897 |
| 525 | 0.28 | 0.000710 | 0.000915 | 0.001470 | 0.010445 |
| 530 | 0.272 | 0.000694 | 0.000835 | 0.001455 | 0.009917 |
| 535 | 0.265 | 0.000694 | 0.000819 | 0.001396 | 0.009276 |
| 540 | 0.259 | 0.000710 | 0.000770 | 0.001352 | 0.008560 |
| 545 | 0.253 | 0.000694 | 0.000819 | 0.001308 | 0.007749 |
| 550 | 0.246 | 0.000677 | 0.000738 | 0.001308 | 0.006882 |
| 555 | 0.239 | 0.000677 | 0.000770 | 0.001235 | 0.005920 |
| 560 | 0.232 | 0.000644 | 0.000754 | 0.001205 | 0.005166 |
| 565 | 0.225 | 0.000611 | 0.000722 | 0.001132 | 0.004317 |
| 570 | 0.221 | 0.000578 | 0.000738 | 0.001176 | 0.003582 |
| 575 | 0.216 | 0.000595 | 0.000642 | 0.001161 | 0.003017 |
| 580 | 0.212 | 0.000595 | 0.000690 | 0.001102 | 0.002508 |
| 585 | 0.211 | 0.000562 | 0.000674 | 0.001058 | 0.001810 |
| 590 | 0.205 | 0.000528 | 0.000578 | 0.001088 | 0.001584 |
| 595 | 0.194 | 0.000578 | 0.000594 | 0.001044 | 0.001282 |
| 600 | 0.184 | 0.000528 | 0.000578 | 0.001014 | 0.001225 |
| 605 | 0.176 | 0.000462 | 0.000578 | 0.000985 | 0.001094 |
| 610 | 0.174 | 0.000462 | 0.000546 | 0.000999 | 0.001018 |
| 615 | 0.177 | 0.000495 | 0.000562 | 0.000955 | 0.001018 |
| 620 | 0.179 | 0.000462 | 0.000514 | 0.000970 | 0.000999 |
| 625 | 0.167 | 0.000545 | 0.000546 | 0.000955 | 0.000924 |
| 630 | 0.156 | 0.000429 | 0.000482 | 0.000838 | 0.000867 |
| 635 | 0.144 | 0.000429 | 0.000417 | 0.000779 | 0.000830 |
| 640 | 0.141 | 0.000413 | 0.000449 | 0.000794 | 0.000792 |
| 645 | 0.16 | 0.000363 | 0.000482 | 0.000911 | 0.000905 |
| 650 | 0.182 | 0.000380 | 0.000674 | 0.001073 | 0.001075 |
| 655 | 0.188 | 0.000495 | 0.000642 | 0.001102 | 0.001131 |
| 660 | 0.2 | 0.000562 | 0.000754 | 0.001176 | 0.001244 |
| 665 | 0.212 | 0.000595 | 0.000722 | 0.001235 | 0.001225 |
| 670 | 0.214 | 0.000677 | 0.000803 | 0.001249 | 0.001320 |

| | | | | | |
|---|---|---|---|---|---|
| 675 | 0.206 | 0.000628 | 0.000738 | 0.001235 | 0.001207 |
| 680 | 0.202 | 0.000644 | 0.000690 | 0.001220 | 0.001263 |
| 685 | 0.22 | 0.000595 | 0.000722 | 0.001264 | 0.001244 |
| 690 | 0.212 | 0.000628 | 0.000706 | 0.001161 | 0.001225 |
| 695 | 0.168 | 0.000611 | 0.000514 | 0.001029 | 0.001131 |
| 700 | 0.151 | 0.000446 | 0.000562 | 0.000941 | 0.001075 |

**Table S5**. Photovoltaic data of all the LSCs fabricated. The results reported are average values of three experiments. The concentration of chromophores has been the same in all the LSCs to allow for the comparison

| Samples | $J_{sc}$ (mA $cm^{-2}$) | $V_{oc}$ (V) | FF | PCE% | $\eta_{op}$ % | $EDE_{max}$% (at λ/ nm) |
|---|---|---|---|---|---|---|
| LSC 1 | 0.298 | 0.384 | 0.61 | 0.071 | 0.85 | 5.30 (460) |
| LSC 2 | 0.573 | 0.398 | 0.62 | 0.142 | 1.86 | 10.25 (445) |
| LSC 3 | 0.879 | 0.401 | 0.63 | 0.221 | 2.76 | 9.26 (505) |

**J-V curve measurements**

The current–voltage characteristics were recorded using an AM 1.5G solar simulator as the light source and a Keithley 236 source-measure unit. The corresponding fill factor values are listed in Table S4.

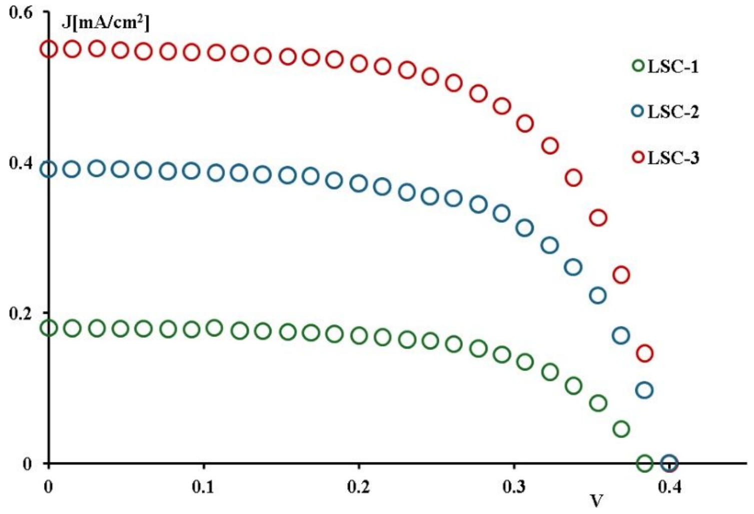


**Figure S4.** J-V curves of the LSC systems

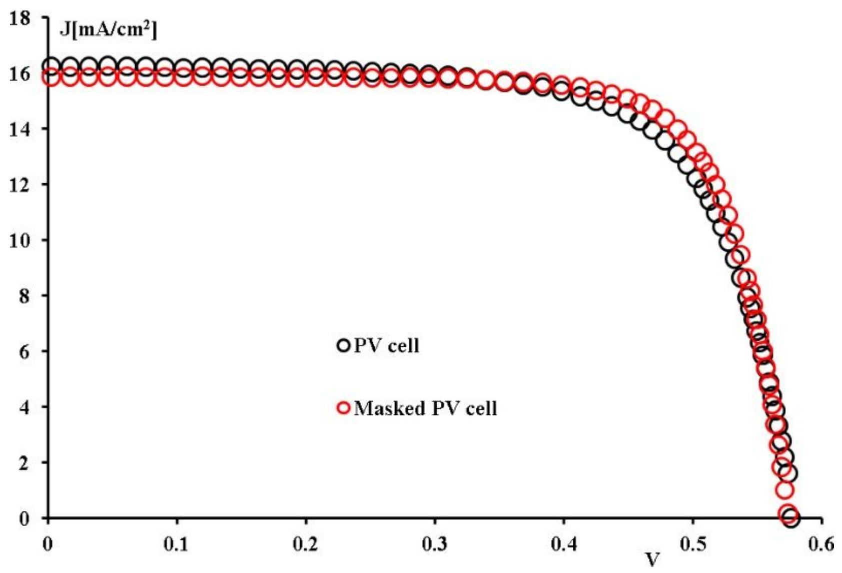


**Figure S5.** J-V curves of the PV cell and masked PV cell

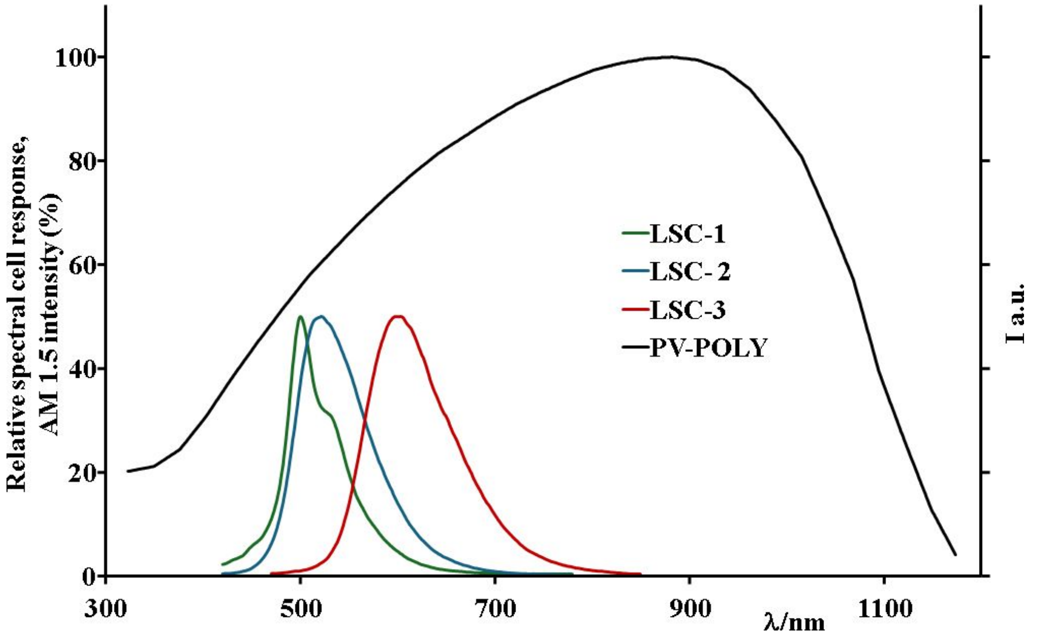


**Figure S6**. Overlap between the emission spectra of the LSCs and the absorption spectrum of the polycrystalline silicon photovoltaic panel.

**NMR SPECTRA**

1

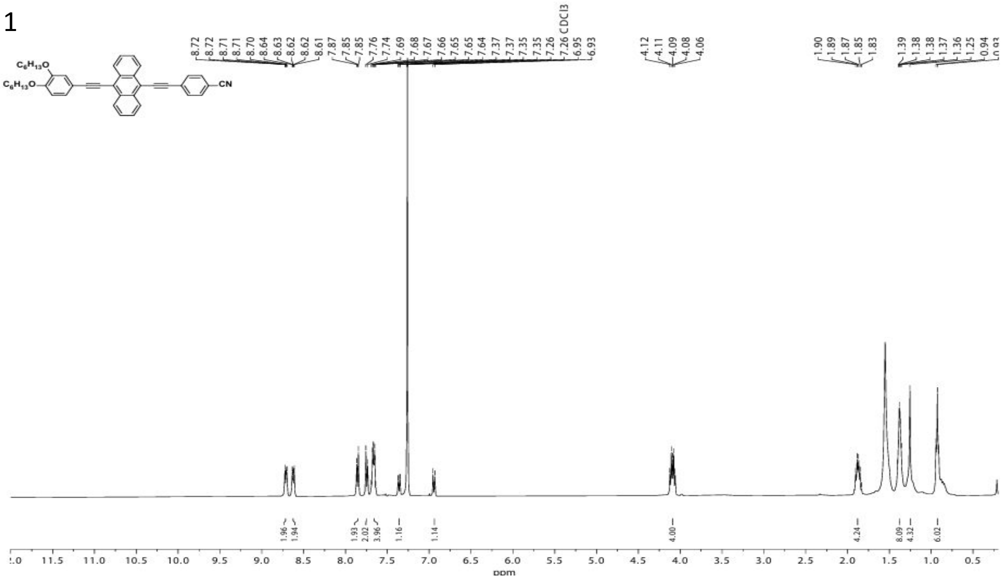

**Figure S7.** $^{1}$H-NMR spectra of compound 1

1

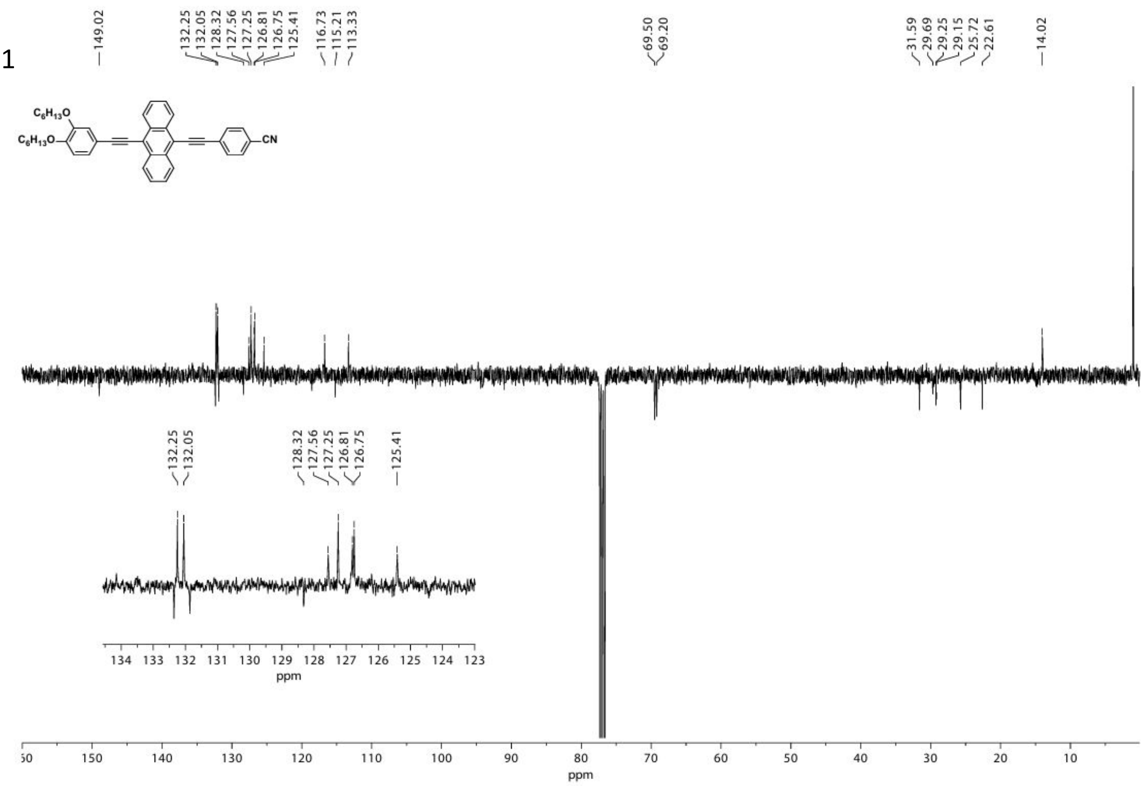

**Figure S8.** $^{13}$C-NMR spectra of compound 1

2

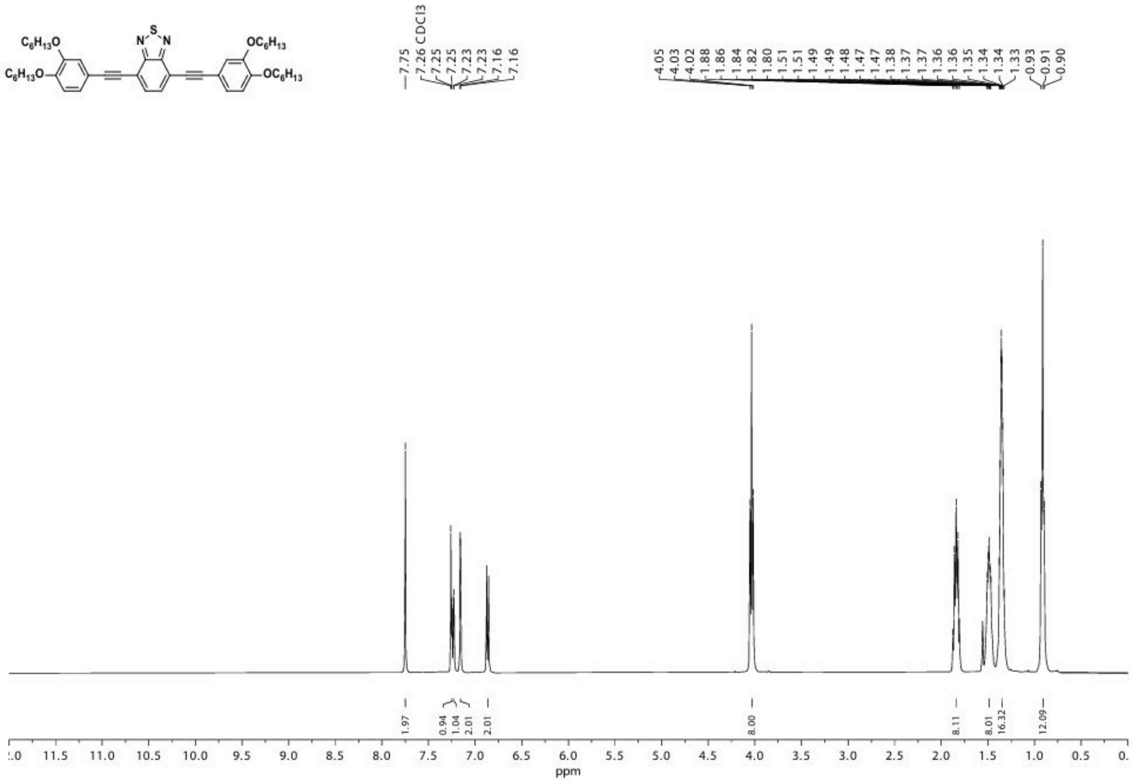


**Figure S9**. $^{1}$H-NMR spectra of compound 2

2

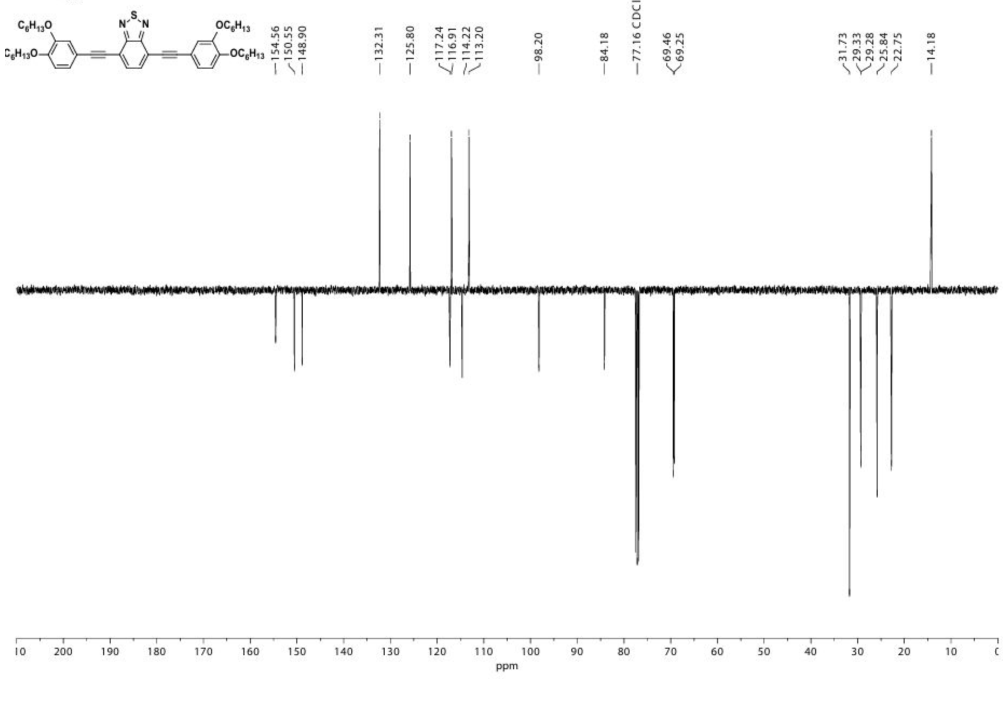

**Figure S10**. $^{13}$C-NMR spectra of compound 2

3

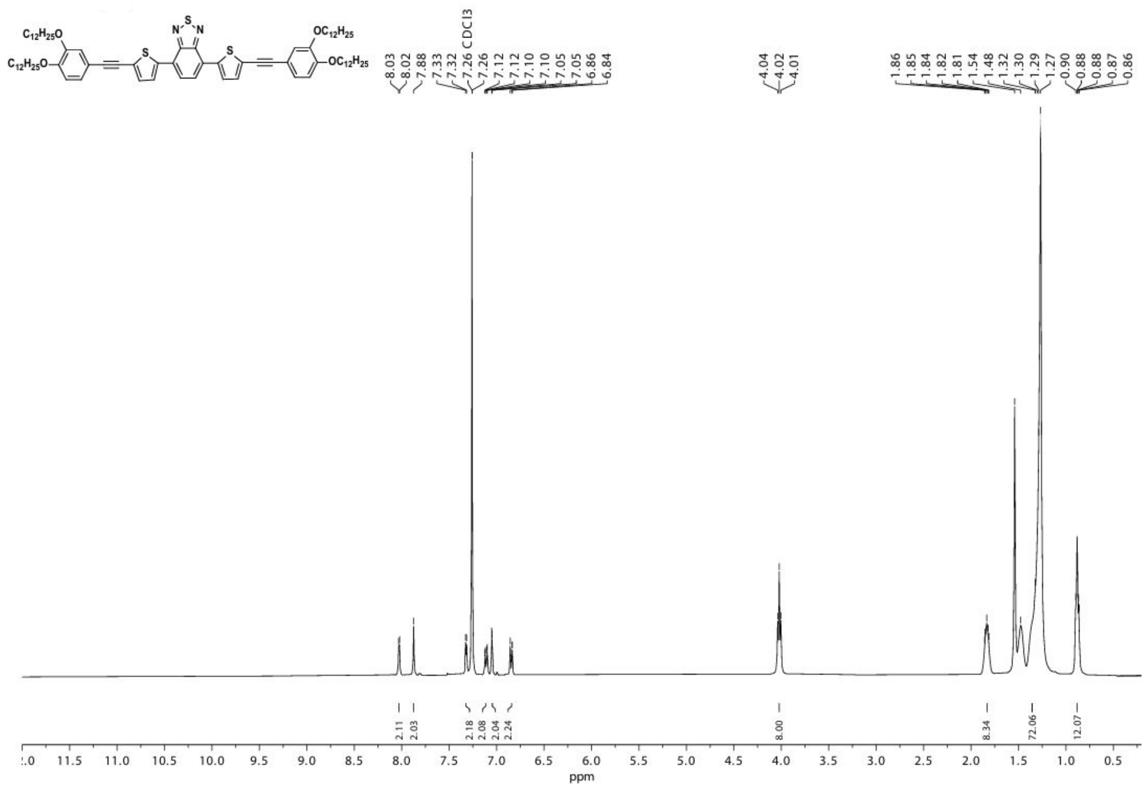


**Figure S11**. $^{1}$H-NMR spectra of compound 3

3

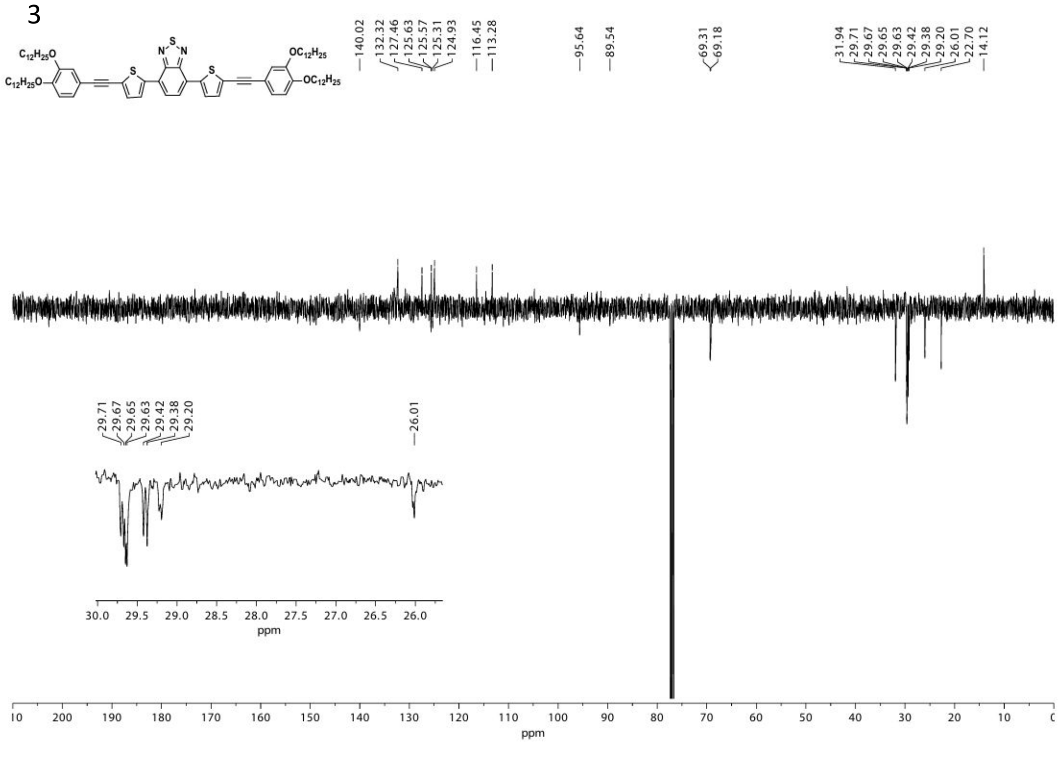

**Figure S12**. $^{13}$C-NMR spectra of compound 3

### AVT, CRI and LAB calculation

Average Visible Transmission (AVT), color coordinates (LAB), Color rendering index (CRI) and Chromaticity Coordinates have been calculated according to the calculator provided in the Supporting Information of the following article: C. Yang, D. Liu, M. Bates, M. C. Barr, R. R. Lunt, "How to Accurately Test, Characterize, and Report Transparent Solar Cells", *Joule,* **2019***, 3,* 1803-1809.[12] Data are summarized in the Tables below.

# LSC-Blank

| Tristimulus Values | AM 1.5G norm. | AM 1.5G*T(λ) norm. |
|---|---|---|
| X | 96.5978 | 96.4976 |
| Y | 100.0000 | 100.0000 |
| Z | 94.2143 | 90.2670 |
| | | |
| Chromaticity Coordinates | AM 1.5G | AM 1.5G*T(λ) |
| x | 0.3322 | 0.3365 |
| y | 0.3439 | 0.3487 |
| z | 0.3240 | 0.3148 |
| u | 0.2056 | 0.2067 |
| v | 0.3193 | 0.3213 |
| | | |
| Uniform Chromaticity Coordinatee (CIELu'v' 1976) | AM 1.5G | AM 1.5G*T(λ) |
| u' | 0.2056 | 0.2067 |
| v' | 0.4789 | 0.4820 |
| CCT estimated (K) | 5513.1846 | 5334.3555 |
| Minimum Chromaticity Distance in CIE1976 (u', v') | 0.0004 | 0.0016 |
| Minimum Chromaticity Distance in CIE1960 (u, v) | 0.0004 | 0.0014 |
| Planckian Limit | 0.0025 | |
| **CRI** | **96.8386** | |
| **AVT%** | **85.6873** | |

| CIELa*b* Coordinates | AM 1.5G ref | AM 1.5G*T(λ) test |
|---|---|---|
| X | 96.5978 | 82.6641 |
| Y | 100.0000 | 85.6644 |
| Z | 94.2143 | 77.3267 |
| f(Xtest/Xref) | 1.0000 | 0.9494 |
| f(Ytest/Yref) | 1.0000 | 0.9497 |
| f(Ztest/Zref) | 1.0000 | 0.9363 |
| L* | 100.0000 | 94.1687 |
| **a*** | **0.0000** | **-0.1642** |
| **b*** | **0.0000** | **2.6907** |

# LSC - 1

| Tristimulus Values | AM 1.5G norm. | AM 1.5G*T(λ) norm. |
|---|---|---|
| X | 96.6 | 96.1 |
| Y | 100.0 | 100.0 |
| Z | 94.2 | 85.3 |

| Chromaticity Coordinates | AM 1.5G | AM 1.5G*T(λ) |
|---|---|---|
| x | 0.332 | 0.341 |
| y | 0.344 | 0.355 |
| z | 0.324 | 0.303 |
| u | 0.206 | 0.207 |
| v | 0.319 | 0.324 |

| Uniform Chromaticity Coordinatee (CIELu'v' 1976) | AM 1.5G | AM 1.5G*T(λ) |
|---|---|---|
| u' | 0.206 | 0.207 |
| v' | 0.479 | 0.486 |
| CCT estimated (K) | 5513 | 5154 |
| Minimum Chromaticity Distance in CIE1976 (u', v') | 0.0004 | 0.0025 |
| Minimum Chromaticity Distance in CIE1960 (u, v) | 0.0004 | 0.0020 |
| Planckian Limit | 0.0025 | |
| **CRI** | **93.57** | |
| **AVT%** | **84.30** | |

| CIELa*b* Coordinates | AM 1.5G ref | AM 1.5G*T(λ) test |
|---|---|---|
| X | 96.6 | 80.9 |
| Y | 100.0 | 84.2 |
| Z | 94.2 | 71.9 |
| f(Xtest/Xref) | 1.00 | 0.94 |
| f(Ytest/Yref) | 1.00 | 0.94 |
| f(Ztest/Zref) | 1.00 | 0.91 |
| L* | 100.0 | 93.6 |
| **a*** | **0.0000** | **-0.86** |

| | | |
|---|---|---|
| **b*** | **0.0000** | **6.12** |

# LSC - 2

| Tristimulus Values | AM 1.5G norm. | AM 1.5G*T(λ) norm. |
|---|---|---|
| X | 96.6 | 95.3 |
| Y | 100.0 | 100.0 |
| Z | 94.2 | 78.1 |

| Chromaticity Coordinates | AM 1.5G | AM 1.5G*T(λ) |
|---|---|---|
| x | 0.332 | 0.349 |
| y | 0.344 | 0.366 |
| z | 0.324 | 0.286 |
| u | 0.206 | 0.208 |
| v | 0.319 | 0.328 |

| Uniform Chromaticity Coordinatee (CIELu'v' 1976) | AM 1.5G | AM 1.5G*T(λ) |
|---|---|---|
| u' | 0.206 | 0.208 |
| v' | 0.479 | 0.492 |
| CCT estimated (K) | 5513 | 4922 |
| Minimum Chromaticity Distance in CIE1976 (u', v') | 0.0004 | 0.0051 |
| Minimum Chromaticity Distance in CIE1960 (u, v) | 0.0004 | 0.0043 |
| Planckian Limit | 0.0025 | |
| **CRI** | **95.14** | |
| **AVT%** | **81.92** | |

| CIELa*b* Coordinates | AM 1.5G ref | AM 1.5G*T(λ) test |
|---|---|---|
| X | 96.6 | 78.0 |
| Y | 100.0 | 81.9 |
| Z | 94.2 | 63.9 |
| f(Xtest/Xref) | 1.00 | 0.93 |
| f(Ytest/Yref) | 1.00 | 0.94 |
| f(Ztest/Zref) | 1.00 | 0.88 |
| L* | 100.0 | 92.5 |
| **a*** | **0.0000** | **-2.15** |
| **b*** | **0.0000** | **11.37** |

# LSC - 3

| Tristimulus Values | AM 1.5G norm. | AM 1.5G*T(λ) norm. |
|---|---|---|
| X | 96.6 | 100.6 |
| Y | 100.0 | 100.0 |
| Z | 94.2 | 90.1 |

| Chromaticity Coordinates | AM 1.5G | AM 1.5G*T(λ) |
|---|---|---|
| x | 0.332 | 0.346 |
| y | 0.344 | 0.344 |
| z | 0.324 | 0.310 |
| u | 0.206 | 0.215 |
| v | 0.319 | 0.321 |

| Uniform Chromaticity Coordinatee (CIELu'v' 1976) | AM 1.5G | AM 1.5G*T(λ) |
|---|---|---|
| u' | 0.206 | 0.215 |
| v' | 0.479 | 0.481 |
| CCT estimated (K) | 5513 | 4945 |
| Minimum Chromaticity Distance in CIE1976 (u', v') | 0.0004 | 0.0067 |
| Minimum Chromaticity Distance in CIE1960 (u, v) | 0.0004 | 0.0056 |
| Planckian Limit | 0.0025 | |
| **CRI** | **93.22** | |
| **AVT%** | **77.57** | |

| CIELa*b* Coordinates | AM 1.5G ref | AM 1.5G*T(λ) test |
|---|---|---|
| X | 96.6 | 77.7 |
| Y | 100.0 | 77.3 |
| Z | 94.2 | 69.6 |
| f(Xtest/Xref) | 1.00 | 0.93 |
| f(Ytest/Yref) | 1.00 | 0.92 |
| f(Ztest/Zref) | 1.00 | 0.90 |
| L* | 100.0 | 90.4 |
| **a*** | **0.0000** | **6.20** |
| **b*** | **0.0000** | **2.71** |

**Table S6** Comparison of the efficiency parameters of LSC–PV devices obtained in this work with those reported in the literature.

| Luminophore | Cell type | PCE [%] | Efficiency Optical | Reference |
| --- | --- | --- | --- | --- |
| LSC 1 | SiPV | 0.07 | 0.85 | |
| LSC 2 | SiPV | 0.14 | 1.86 | |
| LSC 3 | SiPV | 0.22 | 2.76 | |
| Triarylamine1 | SiPV | 0.27 | | 13 |
| Triarylamine2 | SiPV | 0.34 | | 13 |
| Triarylamine3 | SiPV | 0.36 | | 13 |
| PMMA_CD | SiPV | 1.19 | 3.27 | 14 |
| Glass_CD | SiPV | 0.578 | 1.73 | 14 |
| PMMA_Cu_Doped_CD | SiPV | 1.45 | 3.99 | 14 |
| Glass_Cu_Doped_CD | SiPV | 0.705 | 2.11 | 14 |
| Carbon Dots | SiPV | 0.31 | 1.05 | 15 |
| Cdots- Ag40@SiO | SiPV | 0.43 | 1.23 | 15 |
| Cdots | SiPV | | 6.07 | 16 |
| BODIPY–pyrene | SiPV | | 5.67 | 17 |
| pyrene | SiPV | | 6.14 | 17 |
| BODIPY Derivatives | SiPV | | 4.53 | 18 |
| BODIPY Derivatives | SiPV | | 5.26 | 18 |
| BODIPY Derivatives | SiPV | | 8.23 | 18 |
| quantum dots | SiPV | 0.96 | 3.93 | 19 |
| cm | SiPV | 1.24 | 3.02 | 19 |

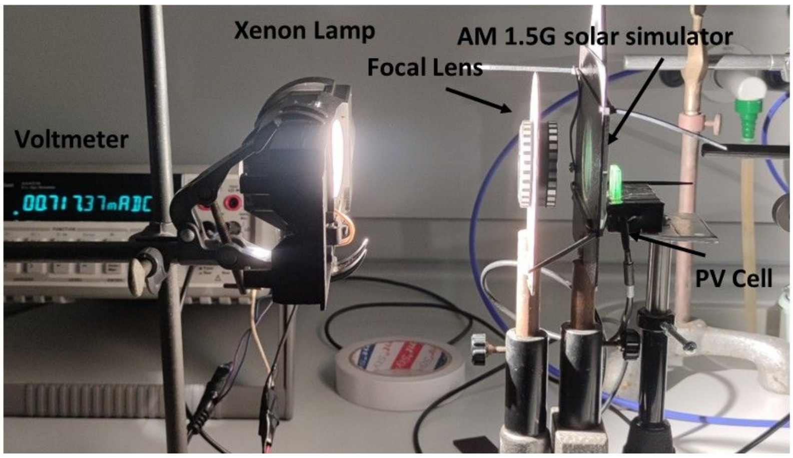


**Figure S13.** Photo of the LSC-PV integration process used**.**

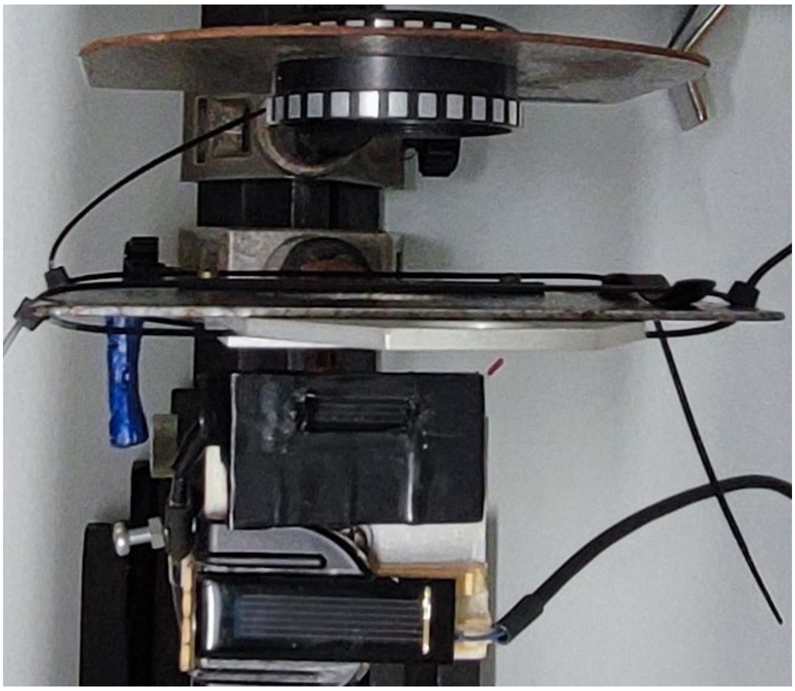

**Figure S14.** Photo of the Pc Cell and masked PV cell (top view).

## REFERENCES


(1) Bartollini, E.; Seri, M.; Tortorella, S.; Facchetti, A.; Marks, T. J.; Marrocchi, A.; Vaccaro, L. Sustainable Synthetic Approach to π-Conjugated Arylacetylenic Semiconductors for Bulk Heterojunction Solar Cells. *RSC Adv.* **2013**, *3* (24), 9288. https://doi.org/10.1039/c3ra41048j.

(2) Silvestri, F.; Marrocchi, A.; Seri, M.; Kim, C.; Marks, T. J.; Facchetti, A.; Taticchi, A. Solution-Processable Low-Molecular Weight Extended Arylacetylenes: Versatile p-Type Semiconductors for Field-Effect Transistors and Bulk Heterojunction Solar Cells. *J. Am. Chem. Soc.* **2010**, *132* (17), 6108–6123. https://doi.org/10.1021/ja910420t.

(3) Marrocchi, A.; Seri, M.; Kim, C.; Facchetti, A.; Taticchi, A.; Marks, T. J. Low-Dimensional Arylacetylenes for Solution-Processable Organic Field-Effect Transistors. *Chem. Mater.* **2009**, *21* (13), 2592–2594. https://doi.org/10.1021/cm900734c.

(4) Wu, Z.; Fan, B.; Xue, F.; Adachi, C.; Ouyang, J. Organic Molecules Based on Dithienyl-2,1,3-Benzothiadiazole as New Donor Materials for Solution-Processed Organic Photovoltaic Cells. *Solar Energy Materials and Solar Cells* **2010**, *94* (12), 2230–2237. https://doi.org/10.1016/j.solmat.2010.07.017.

(5) Mazzaro, R.; Gradone, A.; Angeloni, S.; Morselli, G.; Cozzi, P. G.; Romano, F.; Vomiero, A.; Ceroni, P. Hybrid Silicon Nanocrystals for Color-Neutral and Transparent Luminescent Solar

Concentrators. *ACS Photonics* **2019**, *6* (9), 2303–2311. https://doi.org/10.1021/acsphotonics.9b00802.

(6) Gutierrez, G. D.; Coropceanu, I.; Bawendi, M. G.; Swager, T. M. A Low Reabsorbing Luminescent Solar Concentrator Employing π-Conjugated Polymers. *Advanced Materials* **2016**, *28* (3), 497–501. https://doi.org/10.1002/adma.201504358.

(7) Papakonstantinou, I.; Portnoi, M.; Debije, M. G. The Hidden Potential of Luminescent Solar Concentrators. *Advanced Energy Materials* **2021**, *11* (3), 2002883. https://doi.org/10.1002/aenm.202002883.

(8) Zhou, Y.; Benetti, D.; Tong, X.; Jin, L.; Wang, Z. M.; Ma, D.; Zhao, H.; Rosei, F. Colloidal Carbon Dots Based Highly Stable Luminescent Solar Concentrators. *Nano Energy* **2018**, *44*, 378–387. https://doi.org/10.1016/j.nanoen.2017.12.017.

(9) Arrigo, A.; Cancelliere, A. M.; Galletta, M.; Burtone, A.; Lanteri, G.; Nastasi, F.; Puntoriero, F. From Waste to Energy: Luminescent Solar Concentrators Based on Carbon Dots Derived from Surgical Facemasks. *Mater. Adv.* **2023**, *4* (21), 5200–5205. https://doi.org/10.1039/D3MA00507K.

(10) Cordaro, M.; Neri, G.; Piperno, A.; Cancelliere, A. M.; Santoro, A.; Serroni, S.; Nastasi, F.; Arrigo, A. Comparing a Covalently Linked BODIPY–Pyrene System *versus* the Corresponding Physical Mixture as Chromophores in Luminescent Solar Concentrators. *Sustainable Energy Fuels* **2024**, *8* (10), 2235–2244. https://doi.org/10.1039/D4SE00329B.

(11) Yang, C.; Atwater, H. A.; Baldo, M. A.; Baran, D.; Barile, C. J.; Barr, M. C.; Bates, M.; Bawendi, M. G.; Bergren, M. R.; Borhan, B.; Brabec, C. J.; Brovelli, S.; Bulović, V.; Ceroni, P.; Debije, M. G.; Delgado-Sanchez, J.-M.; Dong, W.-J.; Duxbury, P. M.; Evans, R. C.; Forrest, S. R.; Gamelin, D. R.; Giebink, N. C.; Gong, X.; Griffini, G.; Guo, F.; Herrera, C. K.; Ho-Baillie, A. W. Y.; Holmes, R. J.; Hong, S.-K.; Kirchartz, T.; Levine, B. G.; Li, H.; Li, Y.; Liu, D.; Loi, M. A.; Luscombe, C. K.; Makarov, N. S.; Mateen, F.; Mazzaro, R.; McDaniel, H.; McGehee, M. D.; Meinardi, F.; Menéndez-Velázquez, A.; Min, J.; Mitzi, D. B.; Moemeni, M.; Moon, J. H.; Nattestad, A.; Nazeeruddin, M. K.; Nogueira, A. F.; Paetzold, U. W.; Patrick, D. L.; Pucci, A.; Rand, B. P.; Reichmanis, E.; Richards, B. S.; Roncali, J.; Rosei, F.; Schmidt, T. W.; So, F.; Tu, C.-C.; Vahdani, A.; Van Sark, W. G. J. H. M.; Verduzco, R.; Vomiero, A.; Wong, W. W. H.; Wu, K.; Yip, H.-L.; Zhang, X.; Zhao, H.; Lunt, R. R. Consensus Statement: Standardized Reporting of Power-Producing Luminescent Solar Concentrator Performance. *Joule* **2022**, *6* (1), 8–15. https://doi.org/10.1016/j.joule.2021.12.004.

(12) Li, X.; Ni, G.; Cooper, T.; Xu, N.; Li, J.; Zhou, L.; Hu, X.; Zhu, B.; Yao, P.; Zhu, J. Measuring Conversion Efficiency of Solar Vapor Generation. *Joule* **2019**, *3* (8), 1798–1803. https://doi.org/10.1016/j.joule.2019.06.009.

(13) Tatsi, E.; Raglione, V; Ragno, G. R.; Turri, S.; Mattioli, G.; Porcelli, F.; Caschera, D.; Botta, C.; Zanotti, G.; Griffini, G.; Luminescent solar concentrators based on environmentally friendly tripodal D–(pi–A)3 triarylamine luminophores *J. Mater. Chem. C* **2025** https://doi.org/10.1039/D5TC01559F

(14) Hosseinpanahi K.; Abbaspour-Fard, M. H.; K. Goharshadi, E.; Golzarian, M R.; Sajadi, S.; Vomiero, A.; Enhanced optical properties of luminescent solar concentrators via metal ion doping in carbon dots *J. Mater. Chem.* ***A***, **2025**,**13**, 12639-12649 https://doi.org/10.1039/D5TA00473J

(15) Liu, X.; Benetti, D.; Rosei, F.; Semi-transparent luminescent solar concentrators based on plasmon-enhanced carbon dots *J. Mater. Chem. A*, **2021**, 9, 23345-23352 https://doi.org/10.1039/D1TA02295D

(16) Arrigo, A.; Cancelliere, A. M.; Galletta, M.; Burtone, A.; Lanteri, G.; Nastasi, F.; Puntoriero, F From waste to energy: luminescent solar concentrators based on carbon dots derived from surgical facemasks *Mater. Adv.*, **2023**,4, 5200-5205 https://doi.org/10.1039/D3MA00507K

(17) Cordaro, M.; Neri, G.; Piperno, A.; Cancelliere, A. M.; Santoro, A.; Serroni, S.; Nastasi, F.; Arrigo, A.; Comparing a covalently linked BODIPY–pyrene system versus the corresponding physical mixture as chromophores in luminescent solar concentrators *Sustainable Energy Fuels*, **2024**,8, 2235-2244 https://doi.org/10.1039/D4SE00329B

(18) A. Arrigo, C. M. A. Gangemi, A. Barattucci, P. M. Bonaccorsi, V. Greco, A. Giuffrida, S. Genovese, S. Campagna, F. Nastasi, F. Puntoriero, Highly Efficient Luminescent Solar Concentrators Based on BODIPY Derivatives. *Adv. Mater. Interfaces* **2024**, 11, 2400114. https://doi.org/10.1002/admi.202400114

(19) Choi, J., Kim, K. & Kim, SJ. Quantum dot assisted luminescent hexarhenium cluster dye for a transparent luminescent solar concentrator. *Sci Rep* **11**, 13833 (**2021**). https://doi.org/10.1038/s41598-021-93223-7
